# UMARS: Un-MAppable Reads Solution

**DOI:** 10.1186/1471-2105-12-S1-S9

**Published:** 2011-02-15

**Authors:** Sung-Chou Li, Wen-Ching Chan, Chun-Hung Lai, Kuo-Wang Tsai, Chun-Nan Hsu, Yuh-Shan Jou, Hua-Chien Chen, Chun-Hong Chen, Wen-chang Lin

**Affiliations:** 1 Institute of Biomedical Informatics, National Yang-Ming University, Taipei, Taiwan; 2 Bioinformatics Program, Taiwan International Graduate Program, Academia Sinica, Taipei, Taiwan; 3 Institute of Biomedical Sciences, Academia Sinica, Taipei, Taiwan; 4 Institute of Information Sciences, Academia Sinica, Taipei, Taiwan; 5 Information Sciences Institute, University of Southern California, Marina del Rey, CA 90292, USA; 6 Molecular Medicine Research Center, Chang Gung University, Taoyuan, Taiwan; 7 Division of Molecular and Genomic Medicine, National Health Research Institutes, Zhunan Town, Miaoli County, Taiwan

## Abstract

**Background:**

Un-MAppable Reads Solution (UMARS) is a user-friendly web service focusing on retrieving valuable information from sequence reads that cannot be mapped back to reference genomes. Recently, next-generation sequencing (NGS) technology has emerged as a powerful tool for generating high-throughput sequencing data and has been applied to many kinds of biological research. In a typical analysis, adaptor-trimmed NGS reads were first mapped back to reference sequences, including genomes or transcripts. However, a fraction of NGS reads failed to be mapped back to the reference sequences. Such un-mappable reads are usually imputed to sequencing errors and discarded without further consideration.

**Methods:**

We are investigating possible biological relevance and possible sources of un-mappable reads. Therefore, we developed UMARS to scan for virus genomic fragments or exon-exon junctions of novel alternative splicing isoforms from un-mappable reads. For mapping un-mappable reads, we first collected viral genomes and sequences of exon-exon junctions. Then, we constructed UMARS pipeline as an automatic alignment interface.

**Results:**

By demonstrating the results of two UMARS alignment cases, we show the applicability of UMARS. We first showed that the expected EBV genomic fragments can be detected by UMARS. Second, we also detected exon-exon junctions from un-mappable reads. Further experimental validation also ensured the authenticity of the UMARS pipeline. The UMARS service is freely available to the academic community and can be accessed via http://musk.ibms.sinica.edu.tw/UMARS/.

**Conclusions:**

In this study, we have shown that some un-mappable reads are not caused by sequencing errors. They can originate from viral infection or transcript splicing. Our UMARS pipeline provides another way to examine and recycle the un-mappable reads that are commonly discarded as garbage.

## Background

Biomedical research has been greatly accelerated by the advances in sequencing technologies, especially genomic research. Recently, next-generation sequencing (NGS) technology, including Roche 454, Illumina GA and ABI SOLiD platforms, has emerged as a powerful tool for generating high-throughput sequencing data. Systematic evaluation revealed that these three platforms could possess high sequencing sensitivity because of the large number of reads obtained [1]. Therefore, NGS technology has been applied in many studies, including transcriptome profiling [2-4], SNP identification [5,6], genome sequencing and re-sequencing [7,8], biomarker detection [9], and metagenomics [10,11]. NGS technology was also applied in miRNA identification and profiling studies. Morin and colleagues identified 104 novel human miRNA genes and made a list of miRNAs differentially expressed between embryo cell libraries [12]. Glazov discovered 449 new chicken miRNAs and 39 mirtrons [13]. In addition, Wheeler not only sequenced miRNAs from several metazoan genomes but also studied miRNA’s evolution status [14].

In a typical analysis pipeline, the generated NGS sequence reads are first subject to adaptor trimming and then mapping back to reference sequences, including genomes, scaffolds or transcripts. Several tools, including blast [15], Razers [16], SeqMap [17], SOAP2 [18], BWA [19], MAQ [20] and Bowtie [21], have been used for such mapping. Following the mapping step, the NGS reads are further processed to meet specific experimental interrogations. While it is essential to process the mappable reads in subsequent studies, a fraction of sequence reads cannot be mapped back to reference sequences. In many cases, these un-mappable reads are imputed to sequencing errors and discarded without further consideration. With the rapid increase of NGS reads, we intend to examine the possible biological relevance and possible sources of un-mappable reads. Therefore, we have developed the Un-MAppable Reads Solution (UMARS) pipeline in this study. Although un-mappable reads could originate from platform-specific technique errors, there have been reports demonstrating the possibilities of viral genomic sequences or cryptic splicing isoforms in NGS data [22,23].

Eukaryotic organisms are often infected by different viruses, leading to stable symbiosis or parasitism. As parasites, the infecting viruses rule the infected cells to produce their own genetic materials. Therefore, the collected RNA samples could be contaminated by viral transcripts when tissue or cells are lysed, which produces un-mappable reads when only the host cell genome is used for mapping. Kreuze *et al* detected virus infection by deep sequencing of viral small RNAs [22]. They concluded that NGS technology can be a method for diagnosis and discovery of virus infections. Wu *et al* also reached a similar conclusion [24]. In Kreuze’s study, in addition to the expected infecting viruses, unexpected novel virus reads and unidentified sequence reads also accounted for a large fraction of all reads. The results from these studies demonstrate that the genomic sequences from infecting viruses may contribute to un-mappable reads, and NGS technology is useful for systematic examination of putative viral genomes.

Another possible source of un-mappable reads is cryptic splicing isoforms. During gene expression, eukaryotic genes usually undergo mRNA splicing by removing introns and merging exons. The sequence reads located at the exon-exon junctions of novel alternative splicing isoforms can be mapped back neither to the genome nor to reference mRNAs. For example, Trapnell *et al.* could identify novel wobble splicing junctions from NGS reads [23]. However, there are no specific tools for discovering cryptic alternative splicing exon-exon junctions from large numbers of NGS reads.

At present, there is no biological user-friendly bioinformatic tool or service available focusing on the scanning of viral genomic regions or novel alternative splicing exon-exon junctions from un-mappable reads. We believe that such a tool would be beneficial for biological science researchers.

## Methods and materials

### Collection of genomes and sequences reads

For mapping sequence reads back to viral genomes, we first downloaded 3602 viral genomic sequences from NCBI RefSeq 40 [25]. According to the categories of their hosts, these viral genomes were classified into five classes, including animals, plants, fungi, protozoan plus algae and bacteria plus archaea. We also downloaded the genomic sequences of several animal species from the UCSC genome browser database [26] for extracting exon-exon junctions. The genomic versions of these species are listed in Additional file 1. In this study, sequence reads from NGS technology of several libraries were used. The sequencing platform, RNA source species, and RNA source tissue of these libraries are listed in Additional file 2.

### Extraction of exon-exon junction sequences

**Figure 1:**
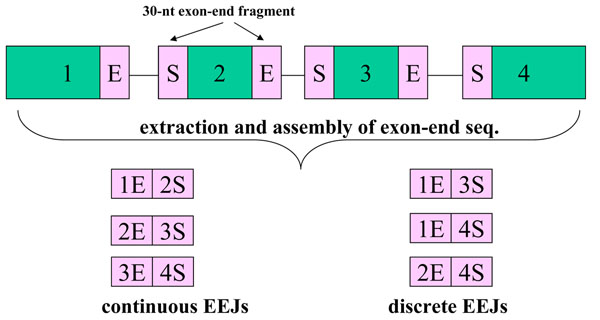
**Collection of exon-exon junctions.** By our definition, the EEJs can be continuous or discrete. The former represent known alternative splicing products. The latter, however, represent novel alternative splicing isoforms.

During maturation, eukaryotic genes usually undergo messenger RNA splicing, producing many alternative splicing isoforms from one gene. UCSC mapped these splicing isoforms back to genomes, and determined the boundaries and genomic coordinates of exons, recording the information in refFlat files. As shown in Fig. 1, from such coordinate information, we may exactly define the boundaries of exons and introns. Further, we may also define the exon-end fragments at exon’s both termini, start (S) or end (E). By extracting and assembling the exon-end fragments, from continuous or discrete exons, we collected 60-nt exon-exon junctions (EEJs). Therefore, they are either continuous or discrete EEJs, where the former denote known splicing patterns and the latter denote novel ones. As a result, the number of 60-nt EEJs is  for each transcript, where *n* is the number of exon in each transcript. By doing so, we collected 60-nt EEJs for 21 species. The refFlat versions, number of EEJs and scientific names of these 21 species are available in Additional file 1.

### Sequence reads processing and mapping

NGS technologies have produced millions of reads, some of which may occur with high frequency. Such high-occurrence reads cause redundancy problems, and should be solved first. Therefore, we developed an in-house tool, called Non-redundant Reads Producer (NRP), to solve this redundancy problem. NRP identifies unique sequence reads from input data, assigned a new ID and tabulates the occurrence frequency (copy number) of each unique read. After NRP processing, non-redundant un-mappable reads may be mapped back to viral genomes or EEJs by UMARS. In the studies involved in mapping sequence reads back to genomes, 100% identity is usually demanded [12,13]. Because viral genomes usually have higher mutation rates than eukaryotic ones, we allowed one nucleotide variation, including mismatch and gap, when mapping back to viral genomes or to EEJ sequences. The mapping procedures in this study were done with blast [15].

### Prediction of viral miRNAs

After the mapping procedure, the viral genomic loci mapped by sequence reads are considered as candidate miRNAs. These genomic loci and their flanking sequences were extracted, followed by alignment using miRNA identification pipeline [27]. For each candidate miRNA, the pipeline first calculated the values of ten features, which serve as discrimination indices in a Support Vector Machine (SVM) algorithm. Then, the SVM was used as a classifier to classify candidate miRNAs into positive or negative sets.

### cDNA preparation

In this study, we used sequence reads from L2 library (Additional file 2) to scan the EEJs of novel alternative splicing isoforms, followed by experimental validation of the detected EEJs in 23 human tissues. Bellow we described how to prepare cDNAs from these tissues. Human tissue poly(A) RNAs (5μg) or total RNAs (40μg) purchased from Clontech (Clontech, Palo Alto, CA) were reverse-transcribed by *Transcriptor* reverse transcriptase (Roche Applied Science), primed by oligo (dT)15 according to the supplier's instructions. After the reverse transcription reaction, the mixtures were phenol-extracted once, followed by chloroform extraction. Excess primers were removed by applying the mixtures to Chroma Spin-200 (Clontech) gel filtration column. The purified cDNAs were properly diluted and subjected to Polymerase Chain Reaction (PCR) as the amplification templates. In this study, cDNAs from 23 tissues were investigated and they were labeled as follow: M: DNA marker, 1: blood, 2: bone marrow, 3: brain, 4: colon, 5: heart, 6: kidney, 7: liver, 8: lung, 9: ovary, 10: pancreas, 11: placenta, 12: skeletal muscle, 13: small intestine, 14: stomach, 15: testis, 16: whole fetus, 17: breast tumor, 18: cervix tumor, 19: colon tumor, 20: kidney tumor, 21: lung tumor, 22: ovary tumor, 23: gastric tumor, 24: PCR no-template control.

### Experimental validation of discrete EEJ

The dtetcted EEJs were verified by PCR amplification, followed by capillary sequencing confirmation. Primer pair sequences were picked from each couple of the “discrete” EEJ-spanning exons and were listed in Additional file 3. PCR components include mainly 1mM dNTP, 1μM primer separately, 0.1U *Takara Taq* DNA polymerase (Takara) per 10μL reaction volume, and the diluted cDNA. The thermal reaction was set at 94°C for 3 minutes, 40 cycles (GAPDH was run specifically for 30 cycles) of denaturing at 94°C for 20 seconds, annealing at 58°C for 30 seconds, and extension at 72°C for 30 seconds, finally at 72°C for 10 minutes. PCR products were separated by 3% NuSieve (Lonza, Rockland, ME) conventional TAE-Agarose gel, and visualized through the ultraviolet light source. The detected and the estimated target size regions of the gel were cut-out and the nucleic-acid contents were purified by Viogene Gel Purification reagents. Minor bands eluted were further subjected to additional 30 PCR cycles with the same pair of primer. The amplified nucleic acid fragments were directly sequenced by ABI 3730xl DNA Analyzer (Applied Biosystems).

## Results and discussion

### UMARS pipeline and interface

**Figure 2:**
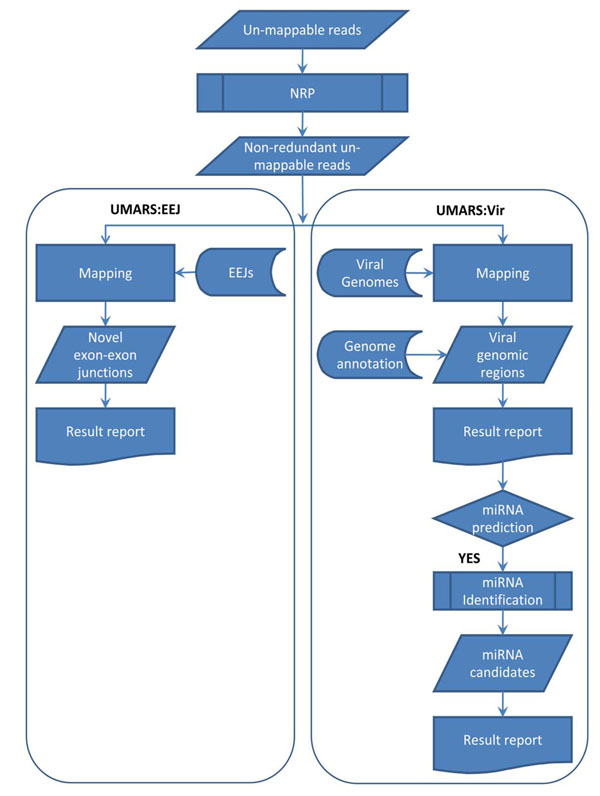
**Flowchart of UMARS service.** The UMARS service can be divided into two subservices: for discovering viral genomic regions (UMARS:Vir); and discovering novel alternative splicing exon-exon junctions (UMARS:EEJ). Un-mappable reads must be processed by NRP to solving redundancy problem before uploaded to UMARS.

The main concept of UMARS is to provide a user-friendly solution for biologists to retrieve valuable information from discarded NGS reads that could not be mapped back to a reference genome. We have initially produced two major datasets for interrogation, a virus genome sequence collection and an animal exon-exon junction sequence collection. Since many NGS studies intend to study miRNAs, we also provided an additional portal for a miRNA discovery pipeline. The overall UMARS schema is illustrated in Fig. 2. The first step of UMARS deals with the redundancy problem of NGS reads. For convenience and efficiency in data processing and network traffic, we developed an in-house tool, called Non-redundant Reads Producer (NRP), to solve the problem. The reads uploaded to UMARS must be processed by NRP in advance or be presented in the format of NRP output. NRP accepts the files containing unmapped read sequences in the FASTA format. An NRP standalone program can be downloaded from the UMARS website. Next, the uploaded non-redundant NGS reads could be further processed by either UMARS:EEJ or UMARS:Vir.

The purpose of UMARS:EEJ is to identify novel alternative splicing exon-exon junctions (EEJs) from un-mappable reads. The sequences of all possible EEJs of 21 species were collected in advance. In UMARS:EEJ, uploaded reads are mapped to EEJs. To avoid random sequence matches, besides our mapping criteria (see Materials and methods), a mapping match must overlap both exons for at least five nucleotides, not skewing too much to either exon. Following the mapping procedure, UMARS tabulats detected EEJs and their expression levels. The detected EEJs are reported as either continuous or discrete EEJs. Continuous EEJs represent known mRNA transcripts. However, discrete EEJs could represent novel splicing isoforms.

The purpose of UMARS:Vir is to identify possible virus genomic regions from un-mappable NGS reads. In UMARS:Vir, the uploaded NGS reads are mapped to all 3,602 known virus genomes. Following the mapping procedure, UMARS tabulates detected virus species and their expression levels. The detected viral genomic regions may locate at intergenic, protein-coding gene, pre-miRNA regions and so on according to the annotations of RefSeq 40 and miRBase 15. Such information of genomic annotation is also provided by the UMARS service. Several viruses are reported to encode viral miRNAs, regulating expression of host genes and playing important roles in host immune misfunctions [28-30]. Therefore, UMARS:Vir may further have the option to detect viral miRNAs by an additional miRNA identification pipeline from viral intergenic genomic regions.

**Figure 3:**
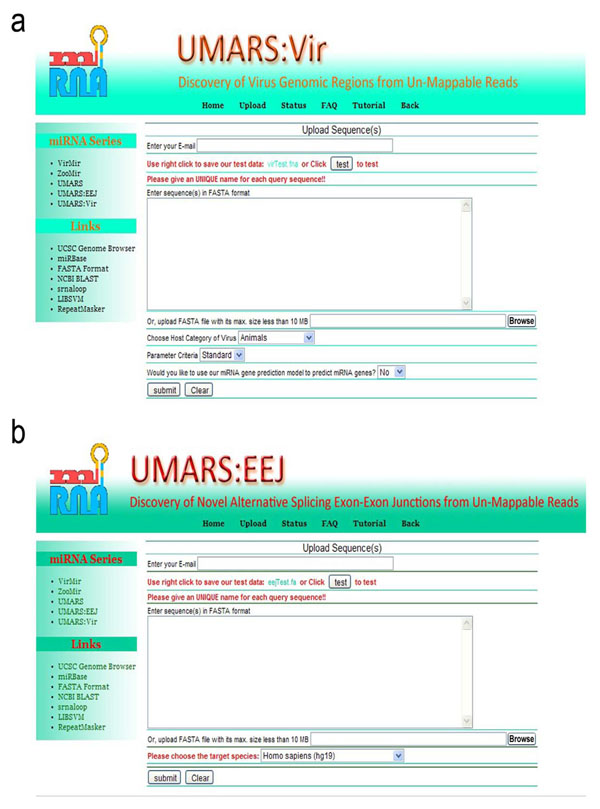
**Interface of UMARS service.** UMARS:Vir and UMARS:EEJ have individual parameters. Users must adjust the parameters according to their specific data source to obtain optimal results. In UMARS:Vir service, users must specify the value, Standard or Loose, of “Parameter Criteria” parameter. Specifying Standard outputs only the viruses with CN >= 100 and RN >= 10 (see Table 1); while specifying Loose outputs all virus with CN >= 1 and RNA >= 1. This is an empirical criterion to reduce random match.

The UMARS service is freely available to the academic community. Users may access UMARS via http://musk.ibms.sinica.edu.tw/UMARS/. The non-redundant reads uploaded into UMARS must not exceed 10 MB in size. Such size limitation has nothing to do with pipeline performance but reduces the network load. As shown in Fig. 3a, in UMARS:Vir, users can select the host category (animals, plants, fungi, protozoan or bacteria) of their expected virus. In UMARS:EEJ, users can select the species corresponding to the EEJs (Fig. 3b). The UMARS results will be sent back to the users via e-mail.

**Table 1:** Summary of viruses detected from L1 un-mappable reads.

Viral acc.	CN	RN	Name	Host
NC_007605.1	105,325 (86,191)	629 (469)	Human herpesvirus 4 type 1	human
NC_006146.1	8,556 (7,311)	157 (34)	Macacine herpesvirus 4	rhesus
NC_001798.1	2,332 (243)	348 (21)	Human herpesvirus 2	human
NC_005261.2	8,247 (200)	304 (18)	Bovine herpesvirus 5	bovine
NC_007653.1	7,458 (172)	363 (24)	Papiine herpesvirus 2	baboon
NC_001806.1	1,773 (147)	278 (13)	Human herpesvirus 1	human
NC_006560.1	6,107 (118)	332 (14)	Cercopithecine herpesvirus 2	monkey
NC_004812.1	5,961 (107)	364 (7)	Macacine herpesvirus 1	rhesus

CN denotes copy number of total reads mappable to the virus; RN denotes the number of distinct genomic regions mapped by reads. The values in parentheses denote the corresponding values when no mismatch was allowed in the mapping procedure. Because their high similarity, most of the reads of type 1 EBV were also mapped back to type 2 EBV.

### Case study and demonstration of UMARS:Vir

To demonstrate the utility of UMARS, we have analyzed NGS reads using UMARS:Vir. In the first case, we investigated the un-mappable reads from the human NPC cells (L1 library) infected with Epstein-Barr virus (EBV, also named human herpes virus 4 type 1). We examined whether the expected EBV genome could be detected by UMARS:Vir. As a result, eight viruses were detected under our mapping criteria. As shown in Table 1, the expected EBV matches dominated over other un-expected viruses in terms of expression level, which shows that UMARS:Vir can be used to detect infections by a specific virus from un-mappable reads. Besides EBV, there were seven un-expected viruses detected, most of which infect primates, and all of them belong to the herpes virus family.

As demonstrated in Table 1, 105,325 reads were mapped back to EBV under our mapping criteria. These EBV reads spanned the EBV genome at 629 genomic regions. The genomic contents, intergenic or intragenic, and detailed information about these regions are listed in Additional file 4. As shown in Table 2 and Additional file 4, 30.1% of these regions are located at the inter-genic regions; 60.1% at the EBV pre-miRNA regions; and only 9.1% at the protein-coding regions. As well, 21 out of 98 protein-coding genes and 22 (excluding mir-BHRF1-1, mir-BHRF1-2, mir-BHRF1-3) out of 25 pre-miRNAs were detected, respectively. This miRNA dominance phenomenon was observed because the L1 sample RNA was extracted with a small RNA kit rather than with a transcriptome kit.

**Table 2:** EBV genomic regions mapped by reads.

Category	Intergenic	Protein coding	pre-miRNA
CN	16,887	1,061	87,788
RN	193	57	379

The regions mapped by reads were classified into three categories according to the annotation of RefSeq 40 and miRBase 15. pre-miRNA regions dominated over other regions in terms of copy number and region number.

Because of the strong sequencing intensity, additional mature miRNAs from the same precursors, including isomiRs at the same arm and minor forms of mature miRNA at the opposite arm, are usually detected from NGS reads [12,13,31]. After arranging miRNA reads in order within their corresponding pre-miRNAs, we observed many isomiRs at all of the 22 detected pre-miRNAs (Additional file 5). Compared with EBV pre-miRNAs in miRBase 15, the reference mature miRNAs do not always represent the most abundant reads. In addition, according to miRBase 15 annotation, mir-BART12, mir-BART16 and mir-BART22 encode mature miRNAs only at their 3’, 5’ and 3’ arm respectively. However, we detected additional mature miRNAs at the 5’ arm of mir-BART12, the 3’ arm of mir-BART16 and the 5’ arm of mir-BART22. Moreover, the 5’ arm of mir-BART12 and the 3’ arm of mir-BART16 encode more reads than the original arms. This result is similar to that in Wheeler’s report [14] and should be noted in future data updates of the miRBase.

### Case study and demonstration of UMARS:EEJ

In addition to virus infections, splicing exon-exon junctions of gene transcripts may also contribute to un-mappable reads. We, therefore, had the un-mappable reads from human H23 cells (L2 library) processed by UMARS:EEJ. As a result, 35,915 un-mappable reads were mapped back to 4,254 EEJs, extracted from 2,738 transcripts (Table 3). On further examination, 70.2% of the detected EEJs were continuous ones, which indicated they were the major mRNA transcription forms.

**Table 3:** Summary of EEJs detected from L2 un-mappable reads.

EEJ category	Continuous	Discrete
**CN**	18,228 (95)	17,687 (1,209)
**# Transcript**	2,038 (14)	700 (69)
**# EEJ**	2,986 (14)	1,269 (83)
**# EST**	581,260 (4,746)	34,469 (1,642)

The digits in parenthesis denote the values when no mismatch was allowed in the mapping procedure. Continuous EEJ dominated over discrete ones in terms of read copy number and EST abundance.

**Figure 4:**
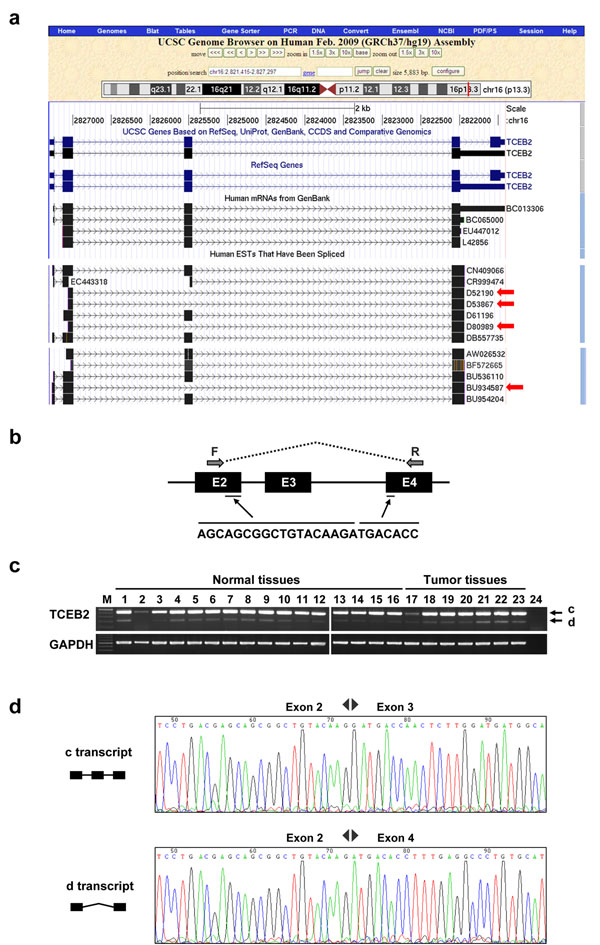
**PCR experimental validation of the detected EEJ, NM_007108 (4:2-4).** The marker lane is labeled with M and is presented as 50bp ladder. The central bright band in the marker lane is equal to 350 bp. (a) UCSC Genome Browser shows that four ESTs match the detected EEJ. (b, c) The PCR result showed that the expected EEJ (d transcript) can be experimentally detected. c and d denotes continuous and discrete transcripts, respectively. (d) The sequencing result provided the authenticity of the detected EEJs.

However, there were 1,269 discrete EEJs detected by UMARS:EEJ from the L2 library. We randomly selected five detected EEJs for PCR validation and three of them were successfully validated. Referring to the UCSC Genome Browser, some discrete EEJs had EST evidence, although these splicing isoforms were not deposited in RefSeq. As shown in Fig. 4a, four ESTs, including BU934587, D80989, D52190 and D53867, matched the detected EEJ from NM_007108 (4:2-4), which was assembled from the exon-end fragments of exon 2 and exon 4. We further designed a PCR experiment in which the forward and reverse primers were located at exon 2 and exon 4, respectively (see Materials and methods). As a result, we succeeded in detecting the expected fragment (d transcript) of the alternative transcript in most of the 23 tissues (Fig. 4b and Fig. 4c), which demonstrated that the EEJ, NM_007108 (4:2-4), occurred almost universally. The sequencing result also proved the authenticity of this discrete transcript (Fig. 4d). Another two detected EEJs, NM_021019(7:2-4) and NM_178580(13:10-13), were also successfully validated (Additional file 6 and Additional file 7).

Table 4 demonstrates the detailed output sample of UMARS:EEJ alignment. The first value “19:6-8” in the “EEJ pattern” column demonstrates that NM_001009566 has 19 exons and the detected EEJ was assembled from the exon-end fragments of exon 6 and exon 8, leading to a discrete EEJ. The first value “14 + 5” in the “Mapping pattern” column demonstrates that the read was split into 14-nt and 5-nt fragments; the former mapped to exon 6 and the latter to exon 8. CN and MM denote the value of the expression level and the number of variations. Because one-nucleotide variation was allowed, three unique reads were mapped back to the same position of the same EEJ. NM_014944 (18:5-7) was also detected owing to alternative splicing from the same gene. All results of our L2 library case are listed in Additional file 8.

**Table 4:** Detected EEJs from CLSTN1.

Gene name	Accession	EEJ pattern	Mapping pattern	Read ID	CN	MM	seq
*CLSTN1*	NM_001009566	19:6-8	14 + 5	NR12	32	0	cctgggtggcaagggtgcg
*CLSTN1*	NM_001009566	19:6-8	14 + 5	NR541	6	1	cctgggtggcatgggtgcg
*CLSTN1*	NM_001009566	19:6-8	14 + 5	NR3652	4	1	cctgggtggcaaggatgcg
*CLSTN1*	NM_014944	18:5-7	14 + 5	NR548	32	0	cctgggtggcaagggtgcg
*CLSTN1*	NM_014944	18:5-7	14 + 5	NR695	6	1	cctgggtggcatgggtgcg
*CLSTN1*	NM_014944	18:5-7	14 + 5	NR47	4	1	cctgggtggcaaggatgcg

NM_001009566 and NM_014944 are splicing isoforms of *CLSTN1*. The EEJ (19:6-8) from NM_001009566 is identical to the EEJ (18:5-7) from NM_014944.

MYL6 (myosin light polypeptide 6) encodes a myosin alkali light chain and is associated with cell migration [32]. It was also reported that fibroblasts promote the growth of breast tumor cells by enhancing the expression of several genes, including MYL6 [33]. In this study, c alternative transcript and d transcript of MYL6 have similar expression levels in most normal tissues (Additional file 6). However, the d transcript dominates over c alternative transcript in most tumor tissues, including breast tumor (17^th^ lane in Additional file 6a). It is possible that these alternative splicing isoforms function differently with each other and are associated with tumor genesis.

## Conclusion

With the rapid increase of sequencing data, UMARS can detect more and more un-expected splicing isoforms which may provide us insights deeper into gene functions and relations to disease. Although NGS technology has been considered a powerful sequencing tool in biological research, large-scale studies, such as those using microarrays, seem to produce un-expected data unavoidably. Such un-expected data could be background noise, and should be eliminated for data accuracy. In this study, we have shown that some un-mappable reads are not caused by sequencing errors. They can originate from viral infection or transcript splicing. Our UMARS pipeline provides another way to examine and recycle the un-mappable reads that are commonly discarded as garbage. Although we have proposed two possible sources for generating un-mappable redas, a fraction of un-mappable reads still failed to be detected by UMARS. More effort should be expended in investigating the biological relevance and possible sources of un-mappable reads.

## Authors’ contributions

SCL and WCC executed this study and wrote the draft of this manuscript. WCC and CNH were responsible for Construction of UMARS interface. CHL did the jobs of cDNA preparation and PCR experiments. YSJ and HCC provided the SOLiD read data. KWT and CHC participated in discussion on research design. WCL supervised the study and edited the manuscript.

## Competing interests

The authors declare that they have no competing interests.

## Supplementary Material

Additional file 1 The refFlat version, genome version, number of transcript, number of exon-exon junction and scientific names of the 21 species. Click here for file

Additional file 2 The sequencing platform, RNA source species, RNA source tissue of these libraries. Click here for file

Additional file 3 Primer sequences involved in this study. Click here for file

Additional file 4 The mapped reads and their corresponding genomic loci. In the Region info column, “-” denoted intergenic regions without known gene annotation. SN. denoted serial numbers of each mapping match. Click here for file

Additional file 5 The detected isomiRs of EBV pre-miRNAs. Click here for file

Additional file 6 **PCR experimental validation of the detected EEJ, NM_021019 (7:2-4).** The description of the marker lane and the abbreviations are the same with the ones of Fig. 4c. The forward and reverse primers were located at exon 2 and exon 4, respectively. (a) The PCR result showed that the expected EEJ (d transcript) can be experimentally detected. Besides, the major c and the minor d transcripts, the c alternative transcript (with 108 bp fewer than c transcript) was also detected. Both of the detected c alternative and d transcript have EST evidences. (b) The sequencing result provided the authenticity of the detected EEJs. Click here for file

Additional file 7 **PCR experimental validation of the detected EEJ, NM_178580 (13:10-13).** The description of the marker lane and the abbreviations are the same with the ones of Fig. 4c. The forward and reverse primers were located at exon 10 and exon 13, respectively. (a) The PCR result showed that the expected EEJ (d2 transcript) can be experimentally detected. A c alternative transcript (with 56 bp more than c transcript) was also detected. In addition the d2 transcript (with exon 11 and 12 skiping), we also detected a d1 transcript (with exon 12 skipping, not originally detected by UMARS). (b) The sequencing result provided the authenticity of the detected EEJs. Click here for file

Additional file 8 The information of all detected discrete EEJs. Values in this table are tab separated and can be opened with Excel. Click here for file
