# Supplementary material for: UMARS: Un-MAppable Reads Solution
Source: BMC Bioinformatics. 2011 Feb 15;12(Suppl 1):S9. doi: 10.1186/1471-2105-12-S1-S9 (PMC3044317; doi:10.1186/1471-2105-12-S1-S9)
Supplement: Additional file 1 — The refFlat version, genome version, number of transcript, number of exon-exon junction and scientific names of the 21 species. [file 1471-2105-12-S1-S9-S1.pdf]

Additional file 1. The refFlat version, genome version, number of transcript, number of exon-exon junction and scientific names of the 21 species.

Species	refFlat and genome version	# transcript	# exon-exon junction
Bos taurus	bosTau4	10,209	522,829
Canis familiaris	canFam2	887	86,265
Caenorhabditis elegans	ce6	24,765	676,470
Ciona intestinalis	ci2	737	39,223
Danio rerio	danRer5	12,934	677,655
Drosophila melanogaster	dm3	20,439	384,887
Equus caballus	equCab2	543	31,149
Felis catus	felCat3	429	5,608
Gallus gallus	galGal3	4,300	321,240
Homo sapiens	hg19	32,912	3,117,553
Mus musculus	mm9	22,243	1,977,516
Monodelphis domestica	monDom4	167	4,407
Ornithorhynchus anatinus	ornAna1	10	29
Oryzias latipes	oryLat2	503	31,736
Pan troglodytes	panTro2	26,467	2,606,523
Pongo abelii	ponAbe2	3,442	233,237
Macaca mulatta	rheMac2	481	16,423
Rattus norvegicus	rn4	14,540	997,871
Strongylocentrotus purpuratus	strPur2	553	36,723
Taeniopygia guttata	taeGut1	77	4,050
Xenopus tropicalis	xenTro2	7,773	367,452
