# Supplementary material for: UMARS: Un-MAppable Reads Solution
Source: BMC Bioinformatics. 2011 Feb 15;12(Suppl 1):S9. doi: 10.1186/1471-2105-12-S1-S9 (PMC3044317; doi:10.1186/1471-2105-12-S1-S9)
Supplement: Additional file 2 — The sequencing platform, RNA source species, RNA source tissue of these libraries. [file 1471-2105-12-S1-S9-S2.pdf]

Additional file 2. The sequencing platform, RNA source species, RNA source tissue of these libraries

**Library ID**	**Sequencing platform**	**RNA source species**	**RNA source tissue**	**Infection virus**
L1	ABI SOLiD	*Homo sapiens*	NPC cells	[a]Human herpesvirus 4 type 1
L2	ABI SOLiD	*Homo sapiens*	H23 cells	[b]-

[a] Virus positive sample

[b] No manual virus infection
