# Supplementary material for: UMARS: Un-MAppable Reads Solution
Source: BMC Bioinformatics. 2011 Feb 15;12(Suppl 1):S9. doi: 10.1186/1471-2105-12-S1-S9 (PMC3044317; doi:10.1186/1471-2105-12-S1-S9)
Supplement: Additional file 3 — Primer sequences involved in this study. [file 1471-2105-12-S1-S9-S3.pdf]

Additional file 3. Primer sequences involved in this study.

**Gene**	**Direction**	**Sequence (5’ -> 3’)**
GAPDH	Forward	TGGTATCGTGGAAGGACTCA
GAPDH	Reverse	AGTGGGTGTCGCTGTTGAAG
TCEB2	Forward	ATCTTCACGGACGCCAAGGAGTCCAG
TCEB2	Reverse	TCACTGCACGGCTTGTTCATTGGCAC
MYL6	Forward	TGTGACTTCACCGAAGACCAGACCGC
MYL6	Reverse	CCAGTGTGACAAGAACATGCCGGAT
HM13	Forward	CTTGAAGAAGAATACCCACACCTACTTC
HM13	Reverse	TGTCACTGCCGCTGGATCCTTAG
