# Supplementary material for: UMARS: Un-MAppable Reads Solution
Source: BMC Bioinformatics. 2011 Feb 15;12(Suppl 1):S9. doi: 10.1186/1471-2105-12-S1-S9 (PMC3044317; doi:10.1186/1471-2105-12-S1-S9)
Supplement: Additional file 4 — The mapped reads and their corresponding genomic loci. In the Region info column, “-” denoted intergenic regions without known gene annotation. SN. denoted serial numbers of each mapping match. [file 1471-2105-12-S1-S9-S4.pdf]

**Additional file 4.** The mapped reads and their corresponding genomic loci. In the Region info column, "-" denoted intergenic regions without known gene annotation. SN. denoted serial numbers of each mapping match.

| SN. | Strand | Start pos. | End pos. | Copy number | Region info | Sequence |
|---|---|---|---|---|---|---|
| 1 | + | 2702 | 2720 | 2 | YP_401633.1; | agggcggcggcccgccgcc |
| 2 | + | 6626 | 6650 | 5 | misc_RNA:product=EBER-1; | ctcaggacctacgctgccctagagg |
| 3 | + | 6627 | 6651 | 10 | misc_RNA:product=EBER-1; | tcaggacctacgctgccctagaggt |
| 4 | + | 6627 | 6651 | 2 | misc_RNA:product=EBER-1; | tcaggacttacgctgccctagaggt |
| 5 | + | 6628 | 6652 | 40 | misc_RNA:product=EBER-1; | caggacctacgctgccctagaggtt |
| 6 | + | 6629 | 6646 | 3 | misc_RNA:product=EBER-1; | aggacctacgctaccta |
| 7 | + | 6629 | 6646 | 2 | misc_RNA:product=EBER-1; | aggacctacgctgaccta |
| 8 | + | 6629 | 6646 | 20 | misc_RNA:product=EBER-1; | aggacctacgctgcccta |
| 9 | + | 6629 | 6647 | 2 | misc_RNA:product=EBER-1; | aggacctacgctaccctag |
| 10 | + | 6629 | 6647 | 173 | misc_RNA:product=EBER-1; | aggacctacgctgccctag |
| 11 | + | 6629 | 6648 | 14 | misc_RNA:product=EBER-1; | aggacctacgctgccctaga |
| 12 | + | 6629 | 6649 | 2 | misc_RNA:product=EBER-1; | aggacctacgctaccctagag |
| 13 | + | 6629 | 6649 | 899 | misc_RNA:product=EBER-1; | aggacctacgctgccctagag |
| 14 | + | 6629 | 6649 | 4 | misc_RNA:product=EBER-1; | aggacctacgctgcgctagag |
| 15 | + | 6629 | 6650 | 4 | misc_RNA:product=EBER-1; | aggacctacgctgccctagagg |
| 16 | + | 6629 | 6651 | 9 | misc_RNA:product=EBER-1; | aggacctacgctgccctagaggt |
| 17 | + | 6629 | 6652 | 96 | misc_RNA:product=EBER-1; | aggacctacgctgccctagaggtt |

| | | | | | | |
|---|---|---|---|---|---|---|
| 18 | + | 6629 | 6653 | 2 | misc_RNA:product=EBER-1; | aggaccaacgctgccctagaggttt |
| 19 | + | 6629 | 6653 | 2 | misc_RNA:product=EBER-1; | aggacccacgctgccctagaggttt |
| 20 | + | 6629 | 6653 | 5 | misc_RNA:product=EBER-1; | aggacctacgatgccctagaggttt |
| 21 | + | 6629 | 6653 | 13 | misc_RNA:product=EBER-1; | aggacctacgccgccctagaggttt |
| 22 | + | 6629 | 6653 | 6 | misc_RNA:product=EBER-1; | aggacctacgctaccctagaggttt |
| 23 | + | 6629 | 6653 | 6 | misc_RNA:product=EBER-1; | aggacctacgctgcactagaggttt |
| 24 | + | 6629 | 6653 | 5 | misc_RNA:product=EBER-1; | aggacctacgctgccccagaggttt |
| 25 | + | 6629 | 6653 | 3 | misc_RNA:product=EBER-1; | aggacctacgctgccctaaaggttt |
| 26 | + | 6629 | 6653 | 8 | misc_RNA:product=EBER-1; | aggacctacgctgccctagaagttt |
| 27 | + | 6629 | 6653 | 8574 | misc_RNA:product=EBER-1; | aggacctacgctgccctagaggttt |
| 28 | + | 6629 | 6653 | 3 | misc_RNA:product=EBER-1; | aggacctacgctgccctagatgttt |
| 29 | + | 6629 | 6653 | 8 | misc_RNA:product=EBER-1; | aggacctacgctgccctaggggttt |
| 30 | + | 6629 | 6653 | 2 | misc_RNA:product=EBER-1; | aggacctacgctgccctataggttt |
| 31 | + | 6629 | 6653 | 3 | misc_RNA:product=EBER-1; | aggacctacgctgccctggaggttt |
| 32 | + | 6629 | 6653 | 2 | misc_RNA:product=EBER-1; | aggacctacgctgccttagaggttt |
| 33 | + | 6629 | 6653 | 6 | misc_RNA:product=EBER-1; | aggacctacgctgctctagaggttt |
| 34 | + | 6629 | 6653 | 3 | misc_RNA:product=EBER-1; | aggacctacgcttccctagaggttt |
| 35 | + | 6629 | 6653 | 10 | misc_RNA:product=EBER-1; | aggacctacgttgccctagaggttt |
| 36 | + | 6629 | 6653 | 4 | misc_RNA:product=EBER-1; | aggacctactctgccctagaggttt |
| 37 | + | 6629 | 6653 | 3 | misc_RNA:product=EBER-1; | aggacctgcgctgccctagaggttt |
| 38 | + | 6629 | 6653 | 2 | misc_RNA:product=EBER-1; | aggacgtacgctgccctagaggttt |
| 39 | + | 6629 | 6653 | 3 | misc_RNA:product=EBER-1; | aggacttacgctgccctagaggttt |

| | | | | | | |
|---|---|---|---|---|---|---|
| 40 | + | 6629 | 6653 | 6 | misc_RNA:product=EBER-1; | aggatctacgctgccctagaggttt |
| 41 | + | 6630 | 6654 | 46 | misc_RNA:product=EBER-1; | ggacctacgctgccctagaggtttt |
| 42 | + | 6631 | 6655 | 3 | misc_RNA:product=EBER-1; | gacctacgctgccctagaggttttg |
| 43 | + | 6635 | 6659 | 4 | misc_RNA:product=EBER-1; | tacgctgccctagaggttttgctag |
| 44 | + | 6636 | 6660 | 5 | misc_RNA:product=EBER-1; | acgctgccctagaggttttgctagg |
| 45 | + | 6638 | 6662 | 2 | misc_RNA:product=EBER-1; | gctgccctagaggttttgctaggga |
| 46 | + | 6639 | 6663 | 4 | misc_RNA:product=EBER-1; | ctgccctagaggttttgctagggag |
| 47 | + | 6677 | 6701 | 2 | misc_RNA:product=EBER-1; | ctgtagccacccgtcccgggtacaa |
| 48 | + | 6681 | 6705 | 4 | misc_RNA:product=EBER-1; | agccacccgtcccgggtacaagtcc |
| 49 | + | 6695 | 6712 | 2 | misc_RNA:product=EBER-1; | ggtacaagtcccgagtgg |
| 50 | + | 6704 | 6723 | 3 | misc_RNA:product=EBER-1; | cccgggtggtgaggacggtg |
| 51 | + | 6728 | 6750 | 3 | misc_RNA:product=EBER-1; | tggttgtcttcccagactctgct |
| 52 | + | 6749 | 6768 | 2 | misc_RNA:product=EBER-1; | ctttctgccgtcttcggtca |
| 53 | + | 6752 | 6776 | 2 | misc_RNA:product=EBER-1; | tctgccgtcttcggtcaagtaccag |
| 54 | + | 6753 | 6773 | 2 | misc_RNA:product=EBER-1; | ctgccgtcttcggtcaagtac |
| 55 | + | 6753 | 6775 | 2 | misc_RNA:product=EBER-1; | ctgctgtcttcggtcaagtacca |
| 56 | + | 6754 | 6776 | 2 | misc_RNA:product=EBER-1; | tgccgtcttcggtcaagtaccag |
| 57 | + | 6755 | 6779 | 4 | misc_RNA:product=EBER-1; | gccgtcttcggtcaagtaccagctg |
| 58 | + | 6756 | 6778 | 4 | misc_RNA:product=EBER-1; | ccgtcttcggtcaagtaccagct |
| 59 | + | 6756 | 6780 | 19 | misc_RNA:product=EBER-1; | ccgtcttcggtcaagtaccagctgg |
| 60 | + | 6757 | 6781 | 16 | misc_RNA:product=EBER-1; | cgtcttcggtcaagtaccagctggt |
| 61 | + | 6758 | 6782 | 5 | misc_RNA:product=EBER-1; | gtcttcggtcaagtaccagctggtg |

| | | | | | | |
|---|---|---|---|---|---|---|
| 62 | + | 6760 | 6784 | 2 | misc_RNA:product=EBER-1; | cttcggtcaagtaccagctggtggt |
| 63 | + | 6761 | 6785 | 5 | misc_RNA:product=EBER-1; | ttcggtcaagtaccagctggtggtc |
| 64 | + | 6762 | 6782 | 3 | misc_RNA:product=EBER-1; | tcggtcaagtaccagctggtg |
| 65 | + | 6762 | 6785 | 2 | misc_RNA:product=EBER-1; | tcggtcaagtaccagctggtggtc |
| 66 | + | 6762 | 6786 | 6 | misc_RNA:product=EBER-1; | tcggtcaagtaccagctggtggtcc |
| 67 | + | 6763 | 6782 | 2 | misc_RNA:product=EBER-1; | cggtcaagtaccagctggtg |
| 68 | + | 6763 | 6787 | 7 | misc_RNA:product=EBER-1; | cggtcaagtaccagctggtggtccg |
| 69 | + | 6764 | 6788 | 13 | misc_RNA:product=EBER-1; | ggtcaagtaccagctggtggtccgc |
| 70 | + | 6765 | 6785 | 4 | misc_RNA:product=EBER-1; | gtcaagtaccagctggtggtc |
| 71 | + | 6765 | 6786 | 3 | misc_RNA:product=EBER-1; | gtcaagtaccagctggtggtcc |
| 72 | + | 6765 | 6789 | 6 | misc_RNA:product=EBER-1; | gtcaagtaccagctggtggtccgca |
| 73 | + | 6766 | 6787 | 2 | misc_RNA:product=EBER-1; | tcaagtaccagctggtggtccg |
| 74 | + | 6766 | 6788 | 2 | misc_RNA:product=EBER-1; | tcaagtaccagctggtggtccgc |
| 75 | + | 6766 | 6790 | 3 | misc_RNA:product=EBER-1; | tcaagtaccagctggtggtccgcat |
| 76 | + | 6767 | 6789 | 2 | misc_RNA:product=EBER-1; | caagtaccagctggtggtccgca |
| 77 | + | 6767 | 6791 | 5 | misc_RNA:product=EBER-1; | caagtaccagctggtggtccgcatg |
| 78 | + | 6768 | 6785 | 2 | misc_RNA:product=EBER-1; | aagtaccagctggtggtc |
| 79 | + | 6768 | 6788 | 9 | misc_RNA:product=EBER-1; | aagtaccagctggtggtccgc |
| 80 | + | 6768 | 6789 | 3 | misc_RNA:product=EBER-1; | aagtaccagctggtggtccgca |
| 81 | + | 6768 | 6790 | 2 | misc_RNA:product=EBER-1; | aagtaccagctggtggtccgcat |
| 82 | + | 6768 | 6791 | 17 | misc_RNA:product=EBER-1; | aagtaccagctggtggtccgcatg |
| 83 | + | 6768 | 6792 | 41 | misc_RNA:product=EBER-1; | aagtaccagctggtggtccgcatgt |

| | | | | | | |
|---|---|---|---|---|---|---|
| 84 | + | 6769 | 6788 | 4 | misc_RNA:product=EBER-1; | agtaccagctgatggtccgc |
| 85 | + | 6769 | 6788 | 5 | misc_RNA:product=EBER-1; | agtaccagctggtggtccgc |
| 86 | + | 6769 | 6793 | 5 | misc_RNA:product=EBER-1; | agtaccagctggtggtccgcatgtt |
| 87 | + | 6770 | 6788 | 7 | misc_RNA:product=EBER-1; | gtaccagctggtggtccgc |
| 88 | + | 6770 | 6793 | 5 | misc_RNA:product=EBER-1; | gtaccagctggtggtccgcatgtt |
| 89 | + | 6770 | 6793 | 2 | misc_RNA:product=EBER-1; | gtaccagctggtggtccgtatgtt |
| 90 | + | 6770 | 6794 | 2 | misc_RNA:product=EBER-1; | gtaccagctggtggtccgcaggttt |
| 91 | + | 6770 | 6794 | 20 | misc_RNA:product=EBER-1; | gtaccagctggtggtccgcatgttt |
| 92 | + | 6771 | 6794 | 3 | misc_RNA:product=EBER-1; | taccagctggtggtccgcatgttt |
| 93 | + | 6771 | 6795 | 2 | misc_RNA:product=EBER-1; | taccagctggtggtccgcatgtttt |
| 94 | + | 6772 | 6791 | 2 | misc_RNA:product=EBER-1; | accagctggtggtccgcatg |
| 95 | + | 6772 | 6792 | 5 | misc_RNA:product=EBER-1; | accagctggtggtccgcatgt |
| 96 | + | 6772 | 6794 | 12 | misc_RNA:product=EBER-1; | accagctggtggtccgcatgttt |
| 97 | + | 6773 | 6793 | 3 | misc_RNA:product=EBER-1; | ccagctggtggtccgcatgtt |
| 98 | + | 6773 | 6794 | 7 | misc_RNA:product=EBER-1; | ccagctggtggtccgcatgttt |
| 99 | + | 6775 | 6792 | 6 | misc_RNA:product=EBER-1; | agctggtggtccgcatgt |
| 100 | + | 6775 | 6794 | 12 | misc_RNA:product=EBER-1; | agctggtggtccgcatgttt |
| 101 | + | 6954 | 6977 | 4 | misc_RNA:product=EBER-2; | acaggacagccgttgccctagtgg |
| 102 | + | 6954 | 6978 | 15 | misc_RNA:product=EBER-2; | acaggacagccgttgccctagtggt |
| 103 | + | 6956 | 6973 | 6 | misc_RNA:product=EBER-2; | aggacagccgttgcccta |
| 104 | + | 6956 | 6974 | 10 | misc_RNA:product=EBER-2; | aggacagccgttgccctag |
| 105 | + | 6956 | 6976 | 4 | misc_RNA:product=EBER-2; | aggacagccgttgccctagtg |

| | | | | | | |
|---|---|---|---|---|---|---|
| 106 | + | 6956 | 6977 | 4 | misc_RNA:product=EBER-2; | aggacagccgttgccctagtgg |
| 107 | + | 6956 | 6979 | 2 | misc_RNA:product=EBER-2; | aggacagccgttgccctagtggtt |
| 108 | + | 6956 | 6980 | 2 | misc_RNA:product=EBER-2; | aggacagccgttgccccagtggttt |
| 109 | + | 6956 | 6980 | 1375 | misc_RNA:product=EBER-2; | aggacagccgttgccctagtggttt |
| 110 | + | 6980 | 6998 | 3 | misc_RNA:product=EBER-2; | tcggacgcaccgccaacgc |
| 111 | + | 7105 | 7122 | 2 | misc_RNA:product=EBER-2; | gtattcggcttgtccgct |
| 112 | + | 10221 | 10238 | 2 | - | atttcaccccacccccc |
| 113 | + | 10221 | 10239 | 3 | - | atttcaccccaccccccc |
| 114 | + | 10645 | 10662 | 3 | - | cgggaacgggcacaagaa |
| 115 | + | 10724 | 10742 | 3 | - | gctaaggagggccgccttg |
| 116 | + | 11199 | 11216 | 4 | - | ggatcaattacgccttgc |
| 117 | + | 12011 | 12028 | 2 | - | gtgccagacgccttggcc |
| 118 | + | 15079 | 15100 | 3 | - | acatgtcccagacgccttggcc |
| 119 | + | 15079 | 15100 | 5 | - | acatgtgacagacgccttggcc |
| 120 | + | 15083 | 15100 | 2 | - | gtgccagacgccttggcc |
| 121 | + | 15083 | 15100 | 2 | - | gtgccggacgccttggcc |
| 122 | + | 18151 | 18172 | 3 | - | acatgtcccagacgccttggcc |
| 123 | + | 18151 | 18172 | 5 | - | acatgtgacagacgccttggcc |
| 124 | + | 18155 | 18172 | 2 | - | gtgccagacgccttggcc |
| 125 | + | 18155 | 18172 | 2 | - | gtgccggacgccttggcc |
| 126 | + | 21223 | 21244 | 3 | - | acatgtcccagacgccttggcc |
| 127 | + | 21223 | 21244 | 5 | - | acatgtgacagacgccttggcc |

| | | | | | | |
|---|---|---|---|---|---|---|
| 128 | + | 21227 | 21244 | 2 | - | gtgccagacgccttggcc |
| 129 | + | 21227 | 21244 | 2 | - | gtgccggacgccttggcc |
| 130 | + | 24295 | 24316 | 3 | - | acatgtcccagacgccttggcc |
| 131 | + | 24295 | 24316 | 5 | - | acatgtgacagacgccttggcc |
| 132 | + | 24299 | 24316 | 2 | - | gtgccagacgccttggcc |
| 133 | + | 24299 | 24316 | 2 | - | gtgccggacgccttggcc |
| 134 | + | 27367 | 27388 | 3 | - | acatgtcccagacgccttggcc |
| 135 | + | 27367 | 27388 | 5 | - | acatgtgacagacgccttggcc |
| 136 | + | 27371 | 27388 | 2 | - | gtgccagacgccttggcc |
| 137 | + | 27371 | 27388 | 2 | - | gtgccggacgccttggcc |
| 138 | + | 30439 | 30460 | 3 | - | acatgtcccagacgccttggcc |
| 139 | + | 30439 | 30460 | 5 | - | acatgtgacagacgccttggcc |
| 140 | + | 30443 | 30460 | 2 | - | gtgccagacgccttggcc |
| 141 | + | 30443 | 30460 | 2 | - | gtgccggacgccttggcc |
| 142 | + | 33511 | 33532 | 3 | - | acatgtcccagacgccttggcc |
| 143 | + | 33511 | 33532 | 5 | - | acatgtgacagacgccttggcc |
| 144 | + | 33515 | 33532 | 2 | - | gtgccagacgccttggcc |
| 145 | + | 33515 | 33532 | 2 | - | gtgccggacgccttggcc |
| 146 | + | 35360 | 35377 | 2 | - | ccaagctgcttttattcc |
| 147 | + | 41363 | 41380 | 3 | - | atccataagccccgcctt |
| 148 | + | 44043 | 44060 | 2 | - | agaaggttgggttgggaa |
| 149 | + | 45760 | 45778 | 2 | - | agagggactccaacccccc |

| | | | | | | |
|---|---|---|---|---|---|---|
| 150 | + | 59251 | 59268 | 8 | - | gggcggggaggtcgcctt |
| 151 | + | 59251 | 59269 | 3 | - | gggcagggaggacgccttg |
| 152 | + | 59251 | 59269 | 2 | - | gggcggagaggacgccttg |
| 153 | + | 59251 | 59269 | 2 | - | gggcgggcaggacgccttg |
| 154 | + | 59251 | 59269 | 8 | - | gggcggggaggacgccttg |
| 155 | + | 59251 | 59269 | 7 | - | gggcggggaggtcgccttg |
| 156 | + | 59251 | 59269 | 5 | - | gggcggtgaggacgccttg |
| 157 | + | 59252 | 59269 | 3 | - | ggcgaggaggacgccttg |
| 158 | + | 59375 | 59392 | 5 | - | cgccccttgcccgccttg |
| 159 | + | 67745 | 67763 | 11 | YP_401657.1; | ccggctggccgccttggcc |
| 160 | + | 69070 | 69087 | 2 | YP_401658.1; | aatactgccagcgccttg |
| 161 | + | 70997 | 71014 | 2 | - | ccgcgttctggatcgcct |
| 162 | + | 76493 | 76514 | 2 | YP_401665.1; | gtcattatagccgtcagccatct |
| 163 | + | 108319 | 108337 | 2 | - | cggtgctgttgcgccttgg |
| 164 | + | 115419 | 115436 | 3 | - | gtctcagcagccgccttg |
| 165 | + | 121556 | 121575 | 2 | - | caggcggcccgtcgccttgg |
| 166 | + | 121557 | 121574 | 10 | - | aggcgccccgtccccttg |
| 167 | + | 121557 | 121574 | 3 | - | aggcggcccgtcgccttg |
| 168 | + | 121557 | 121575 | 21 | - | aggcgccccgtccccttgg |
| 169 | + | 121557 | 121575 | 2 | - | aggcggcccgtccccttgg |
| 170 | + | 121557 | 121575 | 170 | - | aggcggcccgtcgccttgg |
| 171 | + | 121558 | 121575 | 60 | - | ggcggcccgtcgccttgg |

| | | | | | | |
|---|---|---|---|---|---|---|
| 172 | + | 123512 | 123529 | 4 | - | tccccggcatctctgcca |
| 173 | + | 123512 | 123530 | 2 | - | tccccggcatctctgccaa |
| 174 | + | 125323 | 125340 | 2 | YP_401698.1; | gggcagggaggccgcctt |
| 175 | + | 134194 | 134211 | 4 | YP_401703.1; | ggaggatgtgcccgcctt |
| 176 | + | 136905 | 136922 | 4 | YP_401704.1;YP_401705.1; | aaggcgaggggctattcc |
| 177 | + | 136905 | 136922 | 4 | YP_401704.1;YP_401705.1; | aaggcggggggatattcc |
| 178 | + | 136905 | 136922 | 26 | YP_401704.1;YP_401705.1; | aaggcggggggccattcc |
| 179 | + | 136905 | 136922 | 2 | YP_401704.1;YP_401705.1; | aaggcggggggctattcc |
| 180 | + | 136905 | 136922 | 28 | YP_401704.1;YP_401705.1; | aaggcggggggggtattcc |
| 181 | + | 136905 | 136922 | 19 | YP_401704.1;YP_401705.1; | aaggcgggggggttattcc |
| 182 | + | 136905 | 136923 | 2 | YP_401704.1;YP_401705.1; | aaggcggggggccattccc |
| 183 | + | 136906 | 136923 | 2 | YP_401704.1;YP_401705.1; | aggcggggggccattccc |
| 184 | + | 138379 | 138398 | 2 | - | acgagtggcagcaggcctaa |
| 185 | + | 138380 | 138397 | 2 | - | cgagtggcagcaagccta |
| 186 | + | 139086 | 139103 | 2 | ebv-mir-BART3; | aacctagtgttagtgttg |
| 187 | + | 139086 | 139103 | 2 | ebv-mir-BART3; | aacctggtgttagtgttg |
| 188 | + | 139086 | 139104 | 2 | ebv-mir-BART3; | aacctagtgttagtgttgt |
| 189 | + | 139086 | 139106 | 2 | ebv-mir-BART3; | aacctagtgttagtattgtgc |
| 190 | + | 139086 | 139106 | 4 | ebv-mir-BART3; | aacctagtgttagtgatgtgc |
| 191 | + | 139086 | 139106 | 5 | ebv-mir-BART3; | aacctagtgttagtggtgtgc |
| 192 | + | 139086 | 139106 | 9 | ebv-mir-BART3; | aacctagtgttagtgtagtgc |
| 193 | + | 139086 | 139106 | 3 | ebv-mir-BART3; | aacctagtgttagtgtcgtgc |

| | | | | | | |
|---|---|---|---|---|---|---|
| 194 | + | 139086 | 139106 | 66 | ebv-mir-BART3; | aacctagtgttagtgttgtgc |
| 195 | + | 139086 | 139107 | 29 | ebv-mir-BART3; | aacctagtgttagtgttgtgct |
| 196 | + | 139086 | 139108 | 2 | ebv-mir-BART3; | aacctagtgttagtgttgtgctg |
| 197 | + | 139087 | 139105 | 10 | ebv-mir-BART3; | acctagtgttagtgttgtg |
| 198 | + | 139087 | 139106 | 2 | ebv-mir-BART3; | acctagtgttagtgtagtgc |
| 199 | + | 139087 | 139106 | 4 | ebv-mir-BART3; | acctagtgttagtgtcgtgc |
| 200 | + | 139087 | 139106 | 2 | ebv-mir-BART3; | acctagtgttagtgttgtgc |
| 201 | + | 139087 | 139107 | 9 | ebv-mir-BART3; | acctagtgttagtgttgtgct |
| 202 | + | 139087 | 139108 | 2 | ebv-mir-BART3; | acctagtgttagtgttgggctg |
| 203 | + | 139087 | 139108 | 4 | ebv-mir-BART3; | acctagtgttagtgttgtgctg |
| 204 | + | 139087 | 139109 | 3 | ebv-mir-BART3; | acctagtgttagtgttgagctgt |
| 205 | + | 139087 | 139109 | 2 | ebv-mir-BART3; | acctagtgttagtgttgcgctgt |
| 206 | + | 139087 | 139109 | 8 | ebv-mir-BART3; | acctagtgttagtgttgtactgt |
| 207 | + | 139087 | 139109 | 12 | ebv-mir-BART3; | acctagtgttagtgttgtgctgt |
| 208 | + | 139087 | 139110 | 2 | ebv-mir-BART3; | acctagtgttagtgttgtgctgta |
| 209 | + | 139123 | 139140 | 9 | ebv-mir-BART3; | gcgcaccactagtcacca |
| 210 | + | 139123 | 139141 | 3 | ebv-mir-BART3; | gcgcaccactagtcaccag |
| 211 | + | 139123 | 139142 | 13 | ebv-mir-BART3; | gcgcaccactagtcaccagg |
| 212 | + | 139123 | 139143 | 6 | ebv-mir-BART3; | gcgcaccactagtcaccaggt |
| 213 | + | 139123 | 139143 | 17 | ebv-mir-BART3; | gcgcaccactagtcagcaggt |
| 214 | + | 139123 | 139144 | 20 | ebv-mir-BART3; | gcgcaccactagtcaccaggtg |
| 215 | + | 139123 | 139145 | 2 | ebv-mir-BART3; | gcgcaccactagtcaccaagtgt |

| | | | | | | |
|---|---|---|---|---|---|---|
| 216 | + | 139123 | 139145 | 12 | ebv-mir-BART3; | gcgcaccactagtcaccaggtgt |
| 217 | + | 139123 | 139146 | 17 | ebv-mir-BART3; | gcgcaccactagtcaccaagtgtc |
| 218 | + | 139123 | 139146 | 9 | ebv-mir-BART3; | gcgcaccactagtcaccagatgtc |
| 219 | + | 139123 | 139146 | 21 | ebv-mir-BART3; | gcgcaccactagtcaccaggtgtc |
| 220 | + | 139123 | 139146 | 3 | ebv-mir-BART3; | gcgcaccactagtcaccatgtgtc |
| 221 | + | 139123 | 139146 | 13 | ebv-mir-BART3; | gcgcaccactagtcaccgggtgtc |
| 222 | + | 139124 | 139141 | 1475 | ebv-mir-BART3; | cgcaccactagtcaccag |
| 223 | + | 139124 | 139141 | 2 | ebv-mir-BART3; | cgcaccactagtccccag |
| 224 | + | 139124 | 139141 | 3 | ebv-mir-BART3; | cgcaccactagtcgccag |
| 225 | + | 139124 | 139142 | 871 | ebv-mir-BART3; | cgcaccactagtcaccagg |
| 226 | + | 139124 | 139142 | 2 | ebv-mir-BART3; | cgcaccactagtcatcagg |
| 227 | + | 139124 | 139143 | 1109 | ebv-mir-BART3; | cgcaccactagtcaccaggt |
| 228 | + | 139124 | 139143 | 2 | ebv-mir-BART3; | cgcaccactagtcactaggt |
| 229 | + | 139124 | 139143 | 4 | ebv-mir-BART3; | cgcaccactagtcagcaggt |
| 230 | + | 139124 | 139143 | 2 | ebv-mir-BART3; | cgcaccactagtcatcaggt |
| 231 | + | 139124 | 139143 | 2 | ebv-mir-BART3; | cgcaccgctagtcaccaggt |
| 232 | + | 139124 | 139144 | 2 | ebv-mir-BART3; | cgcaccactagtcacaaggtg |
| 233 | + | 139124 | 139144 | 1436 | ebv-mir-BART3; | cgcaccactagtcaccaggtg |
| 234 | + | 139124 | 139144 | 21 | ebv-mir-BART3; | cgcaccactagtcacccggtg |
| 235 | + | 139124 | 139144 | 34 | ebv-mir-BART3; | cgcaccactagtcaccgggtg |
| 236 | + | 139124 | 139144 | 9 | ebv-mir-BART3; | cgcaccactagtcacctggtg |
| 237 | + | 139124 | 139144 | 3 | ebv-mir-BART3; | cgcaccactagtcacgaggtg |

| | | | | | | |
|---|---|---|---|---|---|---|
| 238 | + | 139124 | 139144 | 8 | ebv-mir-BART3; | cgcaccactagtcactaggtg |
| 239 | + | 139124 | 139144 | 2 | ebv-mir-BART3; | cgcaccgctagtcaccaggtg |
| 240 | + | 139124 | 139145 | 4 | ebv-mir-BART3; | cgcaccaccagtcaccaggtgt |
| 241 | + | 139124 | 139145 | 77 | ebv-mir-BART3; | cgcaccactagtcaccaagtgt |
| 242 | + | 139124 | 139145 | 34 | ebv-mir-BART3; | cgcaccactagtcaccacgtgt |
| 243 | + | 139124 | 139145 | 1400 | ebv-mir-BART3; | cgcaccactagtcaccaggtgt |
| 244 | + | 139124 | 139145 | 2 | ebv-mir-BART3; | cgcaccactagtcaccatgtgt |
| 245 | + | 139124 | 139145 | 19 | ebv-mir-BART3; | cgcaccactagtcaccgggtgt |
| 246 | + | 139124 | 139145 | 2 | ebv-mir-BART3; | cgcaccactagtcactaggtgt |
| 247 | + | 139124 | 139145 | 2 | ebv-mir-BART3; | cgcaccgctagtcaccaggtgt |
| 248 | + | 139124 | 139146 | 2 | ebv-mir-BART3; | cgcaccactagccaccaggtgtc |
| 249 | + | 139124 | 139146 | 2 | ebv-mir-BART3; | cgcaccactagtcacaaggtgtc |
| 250 | + | 139124 | 139146 | 498 | ebv-mir-BART3; | cgcaccactagtcaccaagtgtc |
| 251 | + | 139124 | 139146 | 43 | ebv-mir-BART3; | cgcaccactagtcaccacgtgtc |
| 252 | + | 139124 | 139146 | 734 | ebv-mir-BART3; | cgcaccactagtcaccagatgtc |
| 253 | + | 139124 | 139146 | 21 | ebv-mir-BART3; | cgcaccactagtcaccagctgtc |
| 254 | + | 139124 | 139146 | 5958 | ebv-mir-BART3; | cgcaccactagtcaccaggtgtc |
| 255 | + | 139124 | 139146 | 55 | ebv-mir-BART3; | cgcaccactagtcaccagttgtc |
| 256 | + | 139124 | 139146 | 125 | ebv-mir-BART3; | cgcaccactagtcaccatgtgtc |
| 257 | + | 139124 | 139146 | 182 | ebv-mir-BART3; | cgcaccactagtcacccggtgtc |
| 258 | + | 139124 | 139146 | 560 | ebv-mir-BART3; | cgcaccactagtcaccgggtgtc |
| 259 | + | 139124 | 139146 | 42 | ebv-mir-BART3; | cgcaccactagtcacctggtgtc |

| | | | | | | |
|---|---|---|---|---|---|---|
| 260 | + | 139124 | 139146 | 2 | ebv-mir-BART3; | cgcaccactagtcactaggtgtc |
| 261 | + | 139124 | 139146 | 5 | ebv-mir-BART3; | cgcaccactagtcgccaggtgtc |
| 262 | + | 139124 | 139146 | 8 | ebv-mir-BART3; | cgcaccattagtcaccaggtgtc |
| 263 | + | 139124 | 139146 | 6 | ebv-mir-BART3; | cgcaccgctagtcaccaggtgtc |
| 264 | + | 139124 | 139146 | 11 | ebv-mir-BART3; | cgcactactagtcaccaggtgtc |
| 265 | + | 139124 | 139146 | 15 | ebv-mir-BART3; | cgcagcactagtcaccaggtgtc |
| 266 | + | 139124 | 139147 | 2 | ebv-mir-BART3; | cgcaccactagtcaccaagtgtca |
| 267 | + | 139124 | 139147 | 7 | ebv-mir-BART3; | cgcaccactagtcaccagctgtca |
| 268 | + | 139124 | 139147 | 3 | ebv-mir-BART3; | cgcaccactagtcaccaggcgtca |
| 269 | + | 139124 | 139147 | 2 | ebv-mir-BART3; | cgcaccactagtcaccaggggtca |
| 270 | + | 139124 | 139147 | 66 | ebv-mir-BART3; | cgcaccactagtcaccaggtgtca |
| 271 | + | 139124 | 139147 | 2 | ebv-mir-BART3; | cgcaccactagtcaccagttgtca |
| 272 | + | 139124 | 139147 | 6 | ebv-mir-BART3; | cgcaccactagtcaccgggtgtca |
| 273 | + | 139124 | 139148 | 138 | ebv-mir-BART3; | cgcaccactagtcaccagatgtcac |
| 274 | + | 139124 | 139148 | 6 | ebv-mir-BART3; | cgcaccactagtcaccagctgtcac |
| 275 | + | 139124 | 139148 | 47 | ebv-mir-BART3; | cgcaccactagtcaccaggagtcac |
| 276 | + | 139124 | 139148 | 6 | ebv-mir-BART3; | cgcaccactagtcaccaggcgtcac |
| 277 | + | 139124 | 139148 | 34 | ebv-mir-BART3; | cgcaccactagtcaccaggggtcac |
| 278 | + | 139124 | 139148 | 20 | ebv-mir-BART3; | cgcaccactagtcaccaggtatcac |
| 279 | + | 139124 | 139148 | 3 | ebv-mir-BART3; | cgcaccactagtcaccaggtctcac |
| 280 | + | 139124 | 139148 | 372 | ebv-mir-BART3; | cgcaccactagtcaccaggtgtcac |
| 281 | + | 139124 | 139148 | 6 | ebv-mir-BART3; | cgcaccactagtcaccagttgtcac |

| | | | | | | |
|---|---|---|---|---|---|---|
| 282 | + | 139124 | 139148 | 51 | ebv-mir-BART3; | cgcaccactagtcacctggtgtcac |
| 283 | + | 139125 | 139142 | 2 | ebv-mir-BART3; | gcaccactagtcaccagg |
| 284 | + | 139125 | 139145 | 2 | ebv-mir-BART3; | gcaccactagtcaccaggtgt |
| 285 | + | 139125 | 139146 | 6 | ebv-mir-BART3; | gcaccactagtcaccaggtgtc |
| 286 | + | 139228 | 139245 | 12 | ebv-mir-BART4; | gacctgatgctgatggtg |
| 287 | + | 139228 | 139245 | 4 | ebv-mir-BART4; | gacctgatgctgcaggtg |
| 288 | + | 139228 | 139245 | 2 | ebv-mir-BART4; | gacctgatgctgcgggtg |
| 289 | + | 139228 | 139245 | 928 | ebv-mir-BART4; | gacctgatgctgctggtg |
| 290 | + | 139228 | 139245 | 5 | ebv-mir-BART4; | gacctgatgctgttggtg |
| 291 | + | 139228 | 139246 | 10 | ebv-mir-BART4; | gacctgatgctgcaggtgt |
| 292 | + | 139228 | 139246 | 6 | ebv-mir-BART4; | gacctgatgctgcgggtgt |
| 293 | + | 139228 | 139246 | 12 | ebv-mir-BART4; | gacctgatgctgctagtgt |
| 294 | + | 139228 | 139246 | 606 | ebv-mir-BART4; | gacctgatgctgctggtgt |
| 295 | + | 139228 | 139247 | 7 | ebv-mir-BART4; | gacctgatgctgctagtgtg |
| 296 | + | 139228 | 139247 | 554 | ebv-mir-BART4; | gacctgatgctgctggtgtg |
| 297 | + | 139228 | 139247 | 5 | ebv-mir-BART4; | gacctgatgctgcttgtgtg |
| 298 | + | 139228 | 139248 | 3 | ebv-mir-BART4; | gacctgatgatgctggtgtgc |
| 299 | + | 139228 | 139248 | 2 | ebv-mir-BART4; | gacctgatgctgcaggtgtgc |
| 300 | + | 139228 | 139248 | 47 | ebv-mir-BART4; | gacctgatgctgctagtgtgc |
| 301 | + | 139228 | 139248 | 39 | ebv-mir-BART4; | gacctgatgctgctgctgtgc |
| 302 | + | 139228 | 139248 | 11 | ebv-mir-BART4; | gacctgatgctgctggcgtgc |
| 303 | + | 139228 | 139248 | 187 | ebv-mir-BART4; | gacctgatgctgctggggtgc |

| | | | | | | |
|---|---|---|---|---|---|---|
| 304 | + | 139228 | 139248 | 1804 | ebv-mir-BART4; | gacctgatgctgctggtgtgc |
| 305 | + | 139228 | 139248 | 16 | ebv-mir-BART4; | gacctgatgctgctgttgtgc |
| 306 | + | 139228 | 139249 | 5 | ebv-mir-BART4; | gacccgatgctgctggtgtgct |
| 307 | + | 139228 | 139249 | 2 | ebv-mir-BART4; | gacctgacgctgctggtgtgct |
| 308 | + | 139228 | 139249 | 5 | ebv-mir-BART4; | gacctgatgctactggtgtgct |
| 309 | + | 139228 | 139249 | 168 | ebv-mir-BART4; | gacctgatgctgctggcgtgct |
| 310 | + | 139228 | 139249 | 262 | ebv-mir-BART4; | gacctgatgctgctggggtgct |
| 311 | + | 139228 | 139249 | 9 | ebv-mir-BART4; | gacctgatgctgctggtctgct |
| 312 | + | 139228 | 139249 | 1927 | ebv-mir-BART4; | gacctgatgctgctggtgtgct |
| 313 | + | 139228 | 139249 | 4 | ebv-mir-BART4; | gacctgatgctgctggtttgct |
| 314 | + | 139228 | 139249 | 2 | ebv-mir-BART4; | gacctgatgctgttggtgtgct |
| 315 | + | 139228 | 139249 | 2 | ebv-mir-BART4; | gacctgatgttgctggtgtgct |
| 316 | + | 139228 | 139250 | 2 | ebv-mir-BART4; | gacctgatgctgctggcgtgctg |
| 317 | + | 139228 | 139250 | 4 | ebv-mir-BART4; | gacctgatgctgctggggtgctg |
| 318 | + | 139228 | 139250 | 3 | ebv-mir-BART4; | gacctgatgctgctggtgagctg |
| 319 | + | 139228 | 139250 | 21 | ebv-mir-BART4; | gacctgatgctgctggtgcgctg |
| 320 | + | 139228 | 139250 | 77 | ebv-mir-BART4; | gacctgatgctgctggtgtgctg |
| 321 | + | 139228 | 139251 | 7 | ebv-mir-BART4; | gacctgatgctgctgctgtgctgt |
| 322 | + | 139228 | 139251 | 10 | ebv-mir-BART4; | gacctgatgctgctggtgtactgt |
| 323 | + | 139228 | 139251 | 13 | ebv-mir-BART4; | gacctgatgctgctggtgtgctgt |
| 324 | + | 139228 | 139251 | 2 | ebv-mir-BART4; | gacctgatgctgctgttgtgctgt |
| 325 | + | 139228 | 139252 | 2 | ebv-mir-BART4; | gacctgatgctgctggggtgctgta |

| | | | | | | |
|---|---|---|---|---|---|---|
| 326 | + | 139228 | 139252 | 8 | ebv-mir-BART4; | gacctgatgctgctggtgtactgta |
| 327 | + | 139228 | 139252 | 20 | ebv-mir-BART4; | gacctgatgctgctggtgtgctgta |
| 328 | + | 139229 | 139248 | 2 | ebv-mir-BART4; | acctgatgctgctggcgtgc |
| 329 | + | 139266 | 139283 | 2 | ebv-mir-BART4; | cacatcacgtaggcacca |
| 330 | + | 139266 | 139284 | 10 | ebv-mir-BART4; | cacatcacgtaggcaccag |
| 331 | + | 139266 | 139286 | 29 | ebv-mir-BART4; | cacatcacgtaggcaccaggt |
| 332 | + | 139266 | 139288 | 9 | ebv-mir-BART4; | cacatcacgtaggcaccaggtgt |
| 333 | + | 139266 | 139289 | 4 | ebv-mir-BART4; | cacatcacgtaggcaccaagtgtc |
| 334 | + | 139266 | 139289 | 6 | ebv-mir-BART4; | cacatcacgtaggcaccaggtgtc |
| 335 | + | 139266 | 139289 | 2 | ebv-mir-BART4; | cacatcacgtaggcaccagttgtc |
| 336 | + | 139266 | 139290 | 4 | ebv-mir-BART4; | cacatcacgtaggcaccaggtgtca |
| 337 | + | 139351 | 139368 | 59 | ebv-mir-BART1; | tcttagtggaagtgacgt |
| 338 | + | 139351 | 139369 | 20 | ebv-mir-BART1; | tcttagtggaagtgacgtg |
| 339 | + | 139351 | 139369 | 4 | ebv-mir-BART1; | tcttagtggaagtgccgtg |
| 340 | + | 139351 | 139370 | 2 | ebv-mir-BART1; | tcttagtggaagtaacgtgc |
| 341 | + | 139351 | 139370 | 95 | ebv-mir-BART1; | tcttagtggaagtgacgtgc |
| 342 | + | 139351 | 139370 | 17 | ebv-mir-BART1; | tcttagtggaagtgccgtgc |
| 343 | + | 139351 | 139370 | 2 | ebv-mir-BART1; | tcttagtggaagtgtcgtgc |
| 344 | + | 139351 | 139371 | 18 | ebv-mir-BART1; | tcttagtggaagtgacgtgct |
| 345 | + | 139351 | 139372 | 4 | ebv-mir-BART1; | tcttagtggaagtgacgagctg |
| 346 | + | 139351 | 139372 | 56 | ebv-mir-BART1; | tcttagtggaagtgacgtgctg |
| 347 | + | 139351 | 139373 | 8 | ebv-mir-BART1; | tcttagtggaagtgacgcgctgt |

| | | | | | | |
|---|---|---|---|---|---|---|
| 348 | + | 139351 | 139373 | 20 | ebv-mir-BART1; | tcttagtggaagtgacgtactgt |
| 349 | + | 139351 | 139373 | 6 | ebv-mir-BART1; | tcttagtggaagtgacgtgctgt |
| 350 | + | 139351 | 139374 | 24 | ebv-mir-BART1; | tcttagtggaagtgacgtactgtg |
| 351 | + | 139351 | 139374 | 23 | ebv-mir-BART1; | tcttagtggaagtgacgtgctgtg |
| 352 | + | 139351 | 139375 | 6 | ebv-mir-BART1; | tcttagtggaagtgacgtgcagtga |
| 353 | + | 139351 | 139375 | 2 | ebv-mir-BART1; | tcttagtggaagtgacgtgcggtga |
| 354 | + | 139351 | 139375 | 15 | ebv-mir-BART1; | tcttagtggaagtgacgtgctgtga |
| 355 | + | 139351 | 139375 | 3 | ebv-mir-BART1; | tcttagtggaagtgacgtgttgtga |
| 356 | + | 139352 | 139370 | 6 | ebv-mir-BART1; | cttagtggaagtgacgtgc |
| 357 | + | 139387 | 139404 | 689 | ebv-mir-BART1; | tagcaccgctatccacta |
| 358 | + | 139387 | 139405 | 188 | ebv-mir-BART1; | tagcaccgctatccactat |
| 359 | + | 139387 | 139405 | 2 | ebv-mir-BART1; | tagcaccgctatctactat |
| 360 | + | 139387 | 139406 | 3 | ebv-mir-BART1; | tagcaccgctatccaatatg |
| 361 | + | 139387 | 139406 | 558 | ebv-mir-BART1; | tagcaccgctatccactatg |
| 362 | + | 139387 | 139406 | 3 | ebv-mir-BART1; | tagcaccgctatccgctatg |
| 363 | + | 139387 | 139406 | 2 | ebv-mir-BART1; | tagcaccgttatccactatg |
| 364 | + | 139387 | 139407 | 3 | ebv-mir-BART1; | tagcaccgctatccacgatgt |
| 365 | + | 139387 | 139407 | 100 | ebv-mir-BART1; | tagcaccgctatccactatgt |
| 366 | + | 139387 | 139408 | 10 | ebv-mir-BART1; | tagcaccactatccactatgtc |
| 367 | + | 139387 | 139408 | 2 | ebv-mir-BART1; | tagcaccgctatcacactatgtc |
| 368 | + | 139387 | 139408 | 23 | ebv-mir-BART1; | tagcaccgctatccaatatgtc |
| 369 | + | 139387 | 139408 | 13 | ebv-mir-BART1; | tagcaccgctatccacaatgtc |

| | | | | | | |
|---|---|---|---|---|---|---|
| 370 | + | 139387 | 139408 | 50 | ebv-mir-BART1; | tagcaccgctatccaccatgtc |
| 371 | + | 139387 | 139408 | 82 | ebv-mir-BART1; | tagcaccgctatccacgatgtc |
| 372 | + | 139387 | 139408 | 1705 | ebv-mir-BART1; | tagcaccgctatccactatgtc |
| 373 | + | 139387 | 139408 | 9 | ebv-mir-BART1; | tagcaccgctatccactctgtc |
| 374 | + | 139387 | 139408 | 29 | ebv-mir-BART1; | tagcaccgctatccactgtgtc |
| 375 | + | 139387 | 139408 | 28 | ebv-mir-BART1; | tagcaccgctatccagtatgtc |
| 376 | + | 139387 | 139408 | 18 | ebv-mir-BART1; | tagcaccgctatccattatgtc |
| 377 | + | 139387 | 139408 | 2 | ebv-mir-BART1; | tagcaccgttatccactatgtc |
| 378 | + | 139387 | 139409 | 2 | ebv-mir-BART1; | tagcaccgctatccactacgtct |
| 379 | + | 139387 | 139409 | 4 | ebv-mir-BART1; | tagcaccgctatccactaggtct |
| 380 | + | 139387 | 139409 | 122 | ebv-mir-BART1; | tagcaccgctatccactatgtct |
| 381 | + | 139387 | 139409 | 8 | ebv-mir-BART1; | tagcaccgctatccactgtgtct |
| 382 | + | 139387 | 139410 | 15 | ebv-mir-BART1; | tagcaccgctatccactatgtctc |
| 383 | + | 139387 | 139411 | 5 | ebv-mir-BART1; | tagcaccgctatccactacgtctcg |
| 384 | + | 139387 | 139411 | 9 | ebv-mir-BART1; | tagcaccgctatccactaggtctcg |
| 385 | + | 139387 | 139411 | 17 | ebv-mir-BART1; | tagcaccgctatccactatatctcg |
| 386 | + | 139387 | 139411 | 11 | ebv-mir-BART1; | tagcaccgctatccactatgactcg |
| 387 | + | 139387 | 139411 | 123 | ebv-mir-BART1; | tagcaccgctatccactatgcctcg |
| 388 | + | 139387 | 139411 | 554 | ebv-mir-BART1; | tagcaccgctatccactatgtctcg |
| 389 | + | 139387 | 139411 | 3 | ebv-mir-BART1; | tagcaccgctatccactctgtctcg |
| 390 | + | 139387 | 139411 | 44 | ebv-mir-BART1; | tagcaccgctatccactgtgtctcg |
| 391 | + | 139387 | 139411 | 4 | ebv-mir-BART1; | tagcaccgctatccactttgtctcg |

| | | | | | | |
|---|---|---|---|---|---|---|
| 392 | + | 139387 | 139411 | 5 | ebv-mir-BART1; | tagcatcgctatccactatgtctcg |
| 393 | + | 139387 | 139412 | 39 | ebv-mir-BART1; | tagcaccgctatccact-tgtctcgc |
| 394 | + | 139388 | 139405 | 2 | ebv-mir-BART1; | agcaccgctatccactat |
| 395 | + | 139388 | 139406 | 5 | ebv-mir-BART1; | agcaccgctatccactatg |
| 396 | + | 139388 | 139408 | 2 | ebv-mir-BART1; | agcaccgctatccaatatgtc |
| 397 | + | 139388 | 139408 | 5 | ebv-mir-BART1; | agcaccgctatccaccatgtc |
| 398 | + | 139388 | 139408 | 54 | ebv-mir-BART1; | agcaccgctatccactatgtc |
| 399 | + | 139388 | 139408 | 2 | ebv-mir-BART1; | agcaccgctatccactctgtc |
| 400 | + | 139388 | 139408 | 3 | ebv-mir-BART1; | agcaccgctatccagtatgtc |
| 401 | + | 139388 | 139409 | 2 | ebv-mir-BART1; | agcaccgctatccactatgtct |
| 402 | + | 139388 | 139412 | 4 | ebv-mir-BART1; | agcaccgctatccactaggtctcgc |
| 403 | + | 139388 | 139412 | 4 | ebv-mir-BART1; | agcaccgctatccactatgcctcgc |
| 404 | + | 139388 | 139412 | 45 | ebv-mir-BART1; | agcaccgctatccactatgtctcgc |
| 405 | + | 139388 | 139413 | 2 | ebv-mir-BART1; | agcaccgctatccact-tgtctcgcc |
| 406 | + | 139410 | 139434 | 3 | - | cgcccgggctatatgtcgccttacc |
| 407 | + | 139553 | 139570 | 130 | ebv-mir-BART15; | gtcagtggttttgtttcc |
| 408 | + | 139553 | 139571 | 94 | ebv-mir-BART15; | gtcagtggttttgtttcct |
| 409 | + | 139553 | 139572 | 23 | ebv-mir-BART15; | gtcagtggttttgtttcctt |
| 410 | + | 139553 | 139573 | 5 | ebv-mir-BART15; | gtcagtggttttgtttacttg |
| 411 | + | 139553 | 139574 | 2 | ebv-mir-BART15; | gtcagtggtcttgtttccttga |
| 412 | + | 139553 | 139574 | 10 | ebv-mir-BART15; | gtcagtggttttgtctccttga |
| 413 | + | 139553 | 139574 | 92 | ebv-mir-BART15; | gtcagtggttttgtttacttga |

| | | | | | | |
|---|---|---|---|---|---|---|
| 414 | + | 139553 | 139574 | 26 | ebv-mir-BART15; | gtcagtggttttgtttcattga |
| 415 | + | 139553 | 139574 | 5 | ebv-mir-BART15; | gtcagtggttttgtttcgttga |
| 416 | + | 139674 | 139693 | 2 | ebv-mir-BART5; | tcaaggtaaatatagctgcc |
| 417 | + | 139674 | 139694 | 2 | ebv-mir-BART5; | tcaaggtgaatatagctgccc |
| 418 | + | 139674 | 139695 | 3 | ebv-mir-BART5; | tcaaggtgaatatagctgccca |
| 419 | + | 139674 | 139696 | 4 | ebv-mir-BART5; | tcaaggtgaatatagctgcccat |
| 420 | + | 139674 | 139697 | 22 | ebv-mir-BART5; | tcaaggtgaatatagctgcccatc |
| 421 | + | 139674 | 139697 | 3 | ebv-mir-BART5; | tcaaggtgaatatagctgctcatc |
| 422 | + | 139674 | 139698 | 5 | ebv-mir-BART5; | tcaaggtgaatatagctgcccatcg |
| 423 | + | 139675 | 139692 | 138 | ebv-mir-BART5; | caaggtgaatatagctgc |
| 424 | + | 139675 | 139693 | 8 | ebv-mir-BART5; | caaggtgaatatagatgcc |
| 425 | + | 139675 | 139693 | 389 | ebv-mir-BART5; | caaggtgaatatagctgcc |
| 426 | + | 139675 | 139693 | 3 | ebv-mir-BART5; | caaggtgaatatagttgcc |
| 427 | + | 139675 | 139693 | 3 | ebv-mir-BART5; | caaggtgaatatggctgcc |
| 428 | + | 139675 | 139694 | 6 | ebv-mir-BART5; | caaggtgaatatagcagccc |
| 429 | + | 139675 | 139694 | 7 | ebv-mir-BART5; | caaggtgaatatagccgccc |
| 430 | + | 139675 | 139694 | 583 | ebv-mir-BART5; | caaggtgaatatagctgccc |
| 431 | + | 139675 | 139695 | 34 | ebv-mir-BART5; | caaggtgaatatagcagccca |
| 432 | + | 139675 | 139695 | 2 | ebv-mir-BART5; | caaggtgaatatagccgccca |
| 433 | + | 139675 | 139695 | 3 | ebv-mir-BART5; | caaggtgaatatagcggccca |
| 434 | + | 139675 | 139695 | 126 | ebv-mir-BART5; | caaggtgaatatagctaccca |
| 435 | + | 139675 | 139695 | 2 | ebv-mir-BART5; | caaggtgaatatagctcccca |

| | | | | | | |
|---|---|---|---|---|---|---|
| 436 | + | 139675 | 139695 | 2129 | ebv-mir-BART5; | caaggtgaatatagctgccca |
| 437 | + | 139675 | 139696 | 35 | ebv-mir-BART5; | caaggtgaatatagctgaccat |
| 438 | + | 139675 | 139696 | 529 | ebv-mir-BART5; | caaggtgaatatagctgcccat |
| 439 | + | 139675 | 139696 | 9 | ebv-mir-BART5; | caaggtgaatatagctgtccat |
| 440 | + | 139675 | 139697 | 3 | ebv-mir-BART5; | caaggcgaatatagctgcccatc |
| 441 | + | 139675 | 139697 | 2 | ebv-mir-BART5; | caaggtaaatatagctgcccatc |
| 442 | + | 139675 | 139697 | 2 | ebv-mir-BART5; | caaggtgaatacagctgcccatc |
| 443 | + | 139675 | 139697 | 4 | ebv-mir-BART5; | caaggtgaatatagccgcccatc |
| 444 | + | 139675 | 139697 | 103 | ebv-mir-BART5; | caaggtgaatatagctgaccatc |
| 445 | + | 139675 | 139697 | 110 | ebv-mir-BART5; | caaggtgaatatagctgcacatc |
| 446 | + | 139675 | 139697 | 3343 | ebv-mir-BART5; | caaggtgaatatagctgcccatc |
| 447 | + | 139675 | 139697 | 71 | ebv-mir-BART5; | caaggtgaatatagctgcgcatc |
| 448 | + | 139675 | 139697 | 37 | ebv-mir-BART5; | caaggtgaatatagctgctcatc |
| 449 | + | 139675 | 139697 | 3 | ebv-mir-BART5; | caaggtgaatatagctggccatc |
| 450 | + | 139675 | 139697 | 43 | ebv-mir-BART5; | caaggtgaatatagctgtccatc |
| 451 | + | 139675 | 139697 | 3 | ebv-mir-BART5; | caaggtgaatgtagctgcccatc |
| 452 | + | 139675 | 139697 | 2 | ebv-mir-BART5; | caaggtgagtatagctgcccatc |
| 453 | + | 139675 | 139698 | 2 | ebv-mir-BART5; | caaggtgaatatagatgcccatcg |
| 454 | + | 139675 | 139698 | 248 | ebv-mir-BART5; | caaggtgaatatagctacccatcg |
| 455 | + | 139675 | 139698 | 11 | ebv-mir-BART5; | caaggtgaatatagctccccatcg |
| 456 | + | 139675 | 139698 | 42 | ebv-mir-BART5; | caaggtgaatatagctgaccatcg |
| 457 | + | 139675 | 139698 | 58 | ebv-mir-BART5; | caaggtgaatatagctgcacatcg |

| | | | | | | |
|---|---|---|---|---|---|---|
| 458 | + | 139675 | 139698 | 46 | ebv-mir-BART5; | caaggtgaatatagctgccaatcg |
| 459 | + | 139675 | 139698 | 1102 | ebv-mir-BART5; | caaggtgaatatagctgcccatcg |
| 460 | + | 139675 | 139698 | 20 | ebv-mir-BART5; | caaggtgaatatagctgccgatcg |
| 461 | + | 139675 | 139698 | 23 | ebv-mir-BART5; | caaggtgaatatagctgcctatcg |
| 462 | + | 139675 | 139698 | 30 | ebv-mir-BART5; | caaggtgaatatagctgcgcatcg |
| 463 | + | 139675 | 139698 | 12 | ebv-mir-BART5; | caaggtgaatatagctgctcatcg |
| 464 | + | 139675 | 139698 | 2 | ebv-mir-BART5; | caaggtgaatatagctggccatcg |
| 465 | + | 139675 | 139698 | 26 | ebv-mir-BART5; | caaggtgaatatagctgtccatcg |
| 466 | + | 139675 | 139698 | 6 | ebv-mir-BART5; | caaggtgaatatagcttcccatcg |
| 467 | + | 139675 | 139698 | 3 | ebv-mir-BART5; | caaggtgtatatagctgcccatcg |
| 468 | + | 139675 | 139698 | 2 | ebv-mir-BART5; | caaggttaatatagctgcccatcg |
| 469 | + | 139675 | 139699 | 22 | ebv-mir-BART5; | caaggtgaatatagctgccaatcga |
| 470 | + | 139675 | 139699 | 922 | ebv-mir-BART5; | caaggtgaatatagctgcccatcga |
| 471 | + | 139675 | 139699 | 13 | ebv-mir-BART5; | caaggtgaatatagctgcccctcga |
| 472 | + | 139675 | 139699 | 22 | ebv-mir-BART5; | caaggtgaatatagctgccgatcga |
| 473 | + | 139675 | 139699 | 18 | ebv-mir-BART5; | caaggtgaatatagctgcctatcga |
| 474 | + | 139676 | 139693 | 54 | ebv-mir-BART5; | aaggtgaatatagctgcc |
| 475 | + | 139676 | 139694 | 4 | ebv-mir-BART5; | aaggtgaatatagcagccc |
| 476 | + | 139676 | 139694 | 2 | ebv-mir-BART5; | aaggtgaatatagccgccc |
| 477 | + | 139676 | 139694 | 178 | ebv-mir-BART5; | aaggtgaatatagctgccc |
| 478 | + | 139676 | 139695 | 9 | ebv-mir-BART5; | aaggtgaatatagcagccca |
| 479 | + | 139676 | 139695 | 2 | ebv-mir-BART5; | aaggtgaatatagccgccca |

| | | | | | | |
|---|---|---|---|---|---|---|
| 480 | + | 139676 | 139695 | 18 | ebv-mir-BART5; | aaggtgaatatagctaccca |
| 481 | + | 139676 | 139695 | 432 | ebv-mir-BART5; | aaggtgaatatagctgccca |
| 482 | + | 139676 | 139696 | 6 | ebv-mir-BART5; | aaggtgaatatagctgaccat |
| 483 | + | 139676 | 139696 | 214 | ebv-mir-BART5; | aaggtgaatatagctgcccat |
| 484 | + | 139676 | 139697 | 36 | ebv-mir-BART5; | aaggtgaatatagctgaccatc |
| 485 | + | 139676 | 139697 | 29 | ebv-mir-BART5; | aaggtgaatatagctgcacatc |
| 486 | + | 139676 | 139697 | 1070 | ebv-mir-BART5; | aaggtgaatatagctgcccatc |
| 487 | + | 139676 | 139697 | 14 | ebv-mir-BART5; | aaggtgaatatagctgcgcatc |
| 488 | + | 139676 | 139697 | 24 | ebv-mir-BART5; | aaggtgaatatagctgctcatc |
| 489 | + | 139676 | 139697 | 2 | ebv-mir-BART5; | aaggtgaatatagctggccatc |
| 490 | + | 139676 | 139698 | 98 | ebv-mir-BART5; | aaggtgaatatagctacccatcg |
| 491 | + | 139676 | 139698 | 2 | ebv-mir-BART5; | aaggtgaatatagctccccatcg |
| 492 | + | 139676 | 139698 | 6 | ebv-mir-BART5; | aaggtgaatatagctgaccatcg |
| 493 | + | 139676 | 139698 | 18 | ebv-mir-BART5; | aaggtgaatatagctgcacatcg |
| 494 | + | 139676 | 139698 | 7 | ebv-mir-BART5; | aaggtgaatatagctgccaatcg |
| 495 | + | 139676 | 139698 | 348 | ebv-mir-BART5; | aaggtgaatatagctgcccatcg |
| 496 | + | 139676 | 139698 | 2 | ebv-mir-BART5; | aaggtgaatatagctgccgatcg |
| 497 | + | 139676 | 139698 | 5 | ebv-mir-BART5; | aaggtgaatatagctgcctatcg |
| 498 | + | 139676 | 139698 | 4 | ebv-mir-BART5; | aaggtgaatatagctgcgcatcg |
| 499 | + | 139676 | 139698 | 2 | ebv-mir-BART5; | aaggtgaatatagctgctcatcg |
| 500 | + | 139676 | 139698 | 4 | ebv-mir-BART5; | aaggtgaatatagcttcccatcg |
| 501 | + | 139676 | 139699 | 15 | ebv-mir-BART5; | aaggtgaatatagctgcccatcga |

| | | | | | | |
|---|---|---|---|---|---|---|
| 502 | + | 139676 | 139700 | 20 | ebv-mir-BART5; | aaggtgaatatagctgccaatcgac |
| 503 | + | 139676 | 139700 | 12 | ebv-mir-BART5; | aaggtgaatatagctgcccaacgac |
| 504 | + | 139676 | 139700 | 21 | ebv-mir-BART5; | aaggtgaatatagctgcccaccgac |
| 505 | + | 139676 | 139700 | 570 | ebv-mir-BART5; | aaggtgaatatagctgcccatcgac |
| 506 | + | 139676 | 139700 | 3 | ebv-mir-BART5; | aaggtgaatatagctgcccctcgac |
| 507 | + | 139676 | 139700 | 2 | ebv-mir-BART5; | aaggtgaatatagctgcccttcgac |
| 508 | + | 139676 | 139700 | 21 | ebv-mir-BART5; | aaggtgaatatagctgccgatcgac |
| 509 | + | 139676 | 139700 | 24 | ebv-mir-BART5; | aaggtgaatatagctgcctatcgac |
| 510 | + | 139677 | 139695 | 2 | ebv-mir-BART5; | aggtgaatatagctgccca |
| 511 | + | 139677 | 139701 | 3 | ebv-mir-BART5; | aggtgaatatagctgcccaccgacg |
| 512 | + | 139677 | 139701 | 11 | ebv-mir-BART5; | aggtgaatatagctgcccatcgacg |
| 513 | + | 139717 | 139735 | 36 | ebv-mir-BART5; | gtgggccgctgttcaccta |
| 514 | + | 139717 | 139736 | 142 | ebv-mir-BART5; | gtgggccgctgttcacctaa |
| 515 | + | 139717 | 139736 | 13 | ebv-mir-BART5; | gtgggccgctgttcccctaa |
| 516 | + | 139717 | 139737 | 43 | ebv-mir-BART5; | gtgggccgctgttcacctaaa |
| 517 | + | 139718 | 139735 | 4 | ebv-mir-BART5; | tgggccgctgttcaccta |
| 518 | + | 139718 | 139736 | 9 | ebv-mir-BART5; | tgggccgctgttcacctaa |
| 519 | + | 139718 | 139737 | 6 | ebv-mir-BART5; | tgggccgctgttcacctaaa |
| 520 | + | 139719 | 139736 | 2 | ebv-mir-BART5; | gggccgctgttcacctaa |
| 521 | + | 139719 | 139737 | 2 | ebv-mir-BART5; | gggccgctgttcacctaaa |
| 522 | + | 139795 | 139812 | 4 | ebv-mir-BART16; | ttagatagagtgggtgtg |
| 523 | + | 139795 | 139813 | 16 | ebv-mir-BART16; | ttagatagagtgggtgtgt |

| | | | | | | |
|---|---|---|---|---|---|---|
| 524 | + | 139795 | 139814 | 4 | ebv-mir-BART16; | ttagatagagtgggtgtgtg |
| 525 | + | 139795 | 139815 | 5 | ebv-mir-BART16; | ttagatagagtgggcgtgtgc |
| 526 | + | 139795 | 139815 | 3 | ebv-mir-BART16; | ttagatagagtgggtatgtgc |
| 527 | + | 139795 | 139815 | 2 | ebv-mir-BART16; | ttagatagagtgggtgagtgc |
| 528 | + | 139795 | 139815 | 2 | ebv-mir-BART16; | ttagatagagtgggtgcgtgc |
| 529 | + | 139795 | 139815 | 11 | ebv-mir-BART16; | ttagatagagtgggtgggtgc |
| 530 | + | 139795 | 139815 | 19 | ebv-mir-BART16; | ttagatagagtgggtgtgtgc |
| 531 | + | 139795 | 139816 | 2 | ebv-mir-BART16; | ttagatagagtgagtgtgtgct |
| 532 | + | 139795 | 139816 | 5 | ebv-mir-BART16; | ttagatagagtgggtgtgtgct |
| 533 | + | 139795 | 139817 | 2 | ebv-mir-BART16; | ttagatagagtgggtgggtgctc |
| 534 | + | 139795 | 139817 | 3 | ebv-mir-BART16; | ttagatagagtgggtgtgtgctc |
| 535 | + | 139795 | 139818 | 2 | ebv-mir-BART16; | ttagatagagtgggtgtgcgctct |
| 536 | + | 139795 | 139819 | 10 | ebv-mir-BART16; | ttagatagagtgggtgtgtgctctt |
| 537 | + | 139796 | 139816 | 2 | ebv-mir-BART16; | tagatagagtgggtgtgtgct |
| 538 | + | 139834 | 139852 | 10 | ebv-mir-BART16; | agatcaccaccctctatcc |
| 539 | + | 139834 | 139853 | 3 | ebv-mir-BART16; | agatcaccaccctcaatcca |
| 540 | + | 139834 | 139853 | 5 | ebv-mir-BART16; | agatcaccaccctcgatcca |
| 541 | + | 139834 | 139853 | 23 | ebv-mir-BART16; | agatcaccaccctctatcca |
| 542 | + | 139834 | 139854 | 5 | ebv-mir-BART16; | agatcaccaccctctacccat |
| 543 | + | 139834 | 139854 | 12 | ebv-mir-BART16; | agatcaccaccctctagccat |
| 544 | + | 139834 | 139854 | 127 | ebv-mir-BART16; | agatcaccaccctctatccat |
| 545 | + | 139834 | 139854 | 21 | ebv-mir-BART16; | agatcaccaccctctctccat |

| | | | | | | |
|---|---|---|---|---|---|---|
| 546 | + | 139834 | 139854 | 3 | ebv-mir-BART16; | agatcaccaccctctttccat |
| 547 | + | 139834 | 139855 | 2 | ebv-mir-BART16; | agatcaccaccctctaaccata |
| 548 | + | 139834 | 139855 | 4 | ebv-mir-BART16; | agatcaccaccctctacccata |
| 549 | + | 139834 | 139855 | 2 | ebv-mir-BART16; | agatcaccaccctctagccata |
| 550 | + | 139834 | 139855 | 17 | ebv-mir-BART16; | agatcaccaccctctatccata |
| 551 | + | 139834 | 139856 | 2 | ebv-mir-BART16; | agatcaccaccctctatccatat |
| 552 | + | 139834 | 139857 | 71 | ebv-mir-BART16; | agatcaccaccctctatccatatc |
| 553 | + | 139835 | 139852 | 8 | ebv-mir-BART16; | gatcaccaccctctatcc |
| 554 | + | 139835 | 139853 | 14 | ebv-mir-BART16; | gatcaccaccctctatcca |
| 555 | + | 139835 | 139854 | 13 | ebv-mir-BART16; | gatcaccaccctctatccat |
| 556 | + | 139835 | 139855 | 2 | ebv-mir-BART16; | gatcaccaccctctaaccata |
| 557 | + | 139835 | 139855 | 3 | ebv-mir-BART16; | gatcaccaccctctatccata |
| 558 | + | 139835 | 139857 | 2 | ebv-mir-BART16; | gatcaccaccctctatacatatc |
| 559 | + | 139835 | 139857 | 58 | ebv-mir-BART16; | gatcaccaccctctatccatatc |
| 560 | + | 139835 | 139857 | 2 | ebv-mir-BART16; | gatctccaccctctatccatatc |
| 561 | + | 139836 | 139853 | 2 | ebv-mir-BART16; | atcaccaccctctatcca |
| 562 | + | 139836 | 139854 | 11 | ebv-mir-BART16; | atcaccaccctctatccat |
| 563 | + | 139836 | 139854 | 3 | ebv-mir-BART16; | atcaccaccctctctccat |
| 564 | + | 139836 | 139855 | 12 | ebv-mir-BART16; | atcaccaccctctatccata |
| 565 | + | 139836 | 139856 | 4 | ebv-mir-BART16; | atcaccaccctctatccatat |
| 566 | + | 139836 | 139857 | 2 | ebv-mir-BART16; | atcaccaccctctatcaatatc |
| 567 | + | 139836 | 139857 | 181 | ebv-mir-BART16; | atcaccaccctctatccatatc |

| | | | | | | |
|---|---|---|---|---|---|---|
| 568 | + | 139836 | 139857 | 3 | ebv-mir-BART16; | atcaccaccctctatgcatatc |
| 569 | + | 139857 | 139875 | 2 | - | cccacaattgataaacctc |
| 570 | + | 139857 | 139878 | 7 | - | cccacaattgataaacccccgc |
| 571 | + | 139857 | 139878 | 18 | - | cccacaattgataaacctccgc |
| 572 | + | 139857 | 139881 | 11 | - | cccacaattgataaacctccgcatg |
| 573 | + | 139915 | 139932 | 18 | ebv-mir-BART17; | taagaggacgcaggcata |
| 574 | + | 139915 | 139933 | 5 | ebv-mir-BART17; | taagaggacgcaagcatac |
| 575 | + | 139915 | 139933 | 4 | ebv-mir-BART17; | taagaggacgcacgcatac |
| 576 | + | 139915 | 139933 | 11 | ebv-mir-BART17; | taagaggacgcagacatac |
| 577 | + | 139915 | 139933 | 41 | ebv-mir-BART17; | taagaggacgcaggcatac |
| 578 | + | 139915 | 139934 | 28 | ebv-mir-BART17; | taagaggacgcaggcataca |
| 579 | + | 139915 | 139935 | 34 | ebv-mir-BART17; | taagaggacgcaggcatacaa |
| 580 | + | 139915 | 139936 | 3 | ebv-mir-BART17; | taagaggacgcaggcatacaag |
| 581 | + | 139916 | 139936 | 2 | ebv-mir-BART17; | aagaggacgcaggcatacaag |
| 582 | + | 139918 | 139935 | 2 | ebv-mir-BART17; | gaggacgcaggcatacaa |
| 583 | + | 139952 | 139975 | 13 | ebv-mir-BART17; | ttgtatgcctggtgtccccttagt |
| 584 | + | 139953 | 139970 | 93 | ebv-mir-BART17; | tgtatgcctggtgtcccc |
| 585 | + | 139953 | 139971 | 102 | ebv-mir-BART17; | tgtatgcctggtgtcccct |
| 586 | + | 139953 | 139972 | 2 | ebv-mir-BART17; | tgtatgcccggtgtcccctt |
| 587 | + | 139953 | 139972 | 79 | ebv-mir-BART17; | tgtatgcctggtgtcccctt |
| 588 | + | 139953 | 139972 | 8 | ebv-mir-BART17; | tgtatgcctggtgtcgcctt |
| 589 | + | 139953 | 139973 | 2 | ebv-mir-BART17; | tgtatgcctggtgtccactta |

| | | | | | | |
|---|---|---|---|---|---|---|
| 590 | + | 139953 | 139973 | 65 | ebv-mir-BART17; | tgtatgcctggtgtcccctta |
| 591 | + | 139953 | 139974 | 2 | ebv-mir-BART17; | tgtacgcctggtgtccccttag |
| 592 | + | 139953 | 139974 | 205 | ebv-mir-BART17; | tgtatgcctggtgtccccttag |
| 593 | + | 139953 | 139975 | 8 | ebv-mir-BART17; | tgtatacctggtgtccccttagt |
| 594 | + | 139953 | 139975 | 5 | ebv-mir-BART17; | tgtatgcatggtgtccccttagt |
| 595 | + | 139953 | 139975 | 22 | ebv-mir-BART17; | tgtatgcctggtgtccccatagt |
| 596 | + | 139953 | 139975 | 8 | ebv-mir-BART17; | tgtatgcctggtgtcccccctagt |
| 597 | + | 139953 | 139975 | 10 | ebv-mir-BART17; | tgtatgcctggtgtccccgtagt |
| 598 | + | 139953 | 139975 | 693 | ebv-mir-BART17; | tgtatgcctggtgtccccttagt |
| 599 | + | 139953 | 139975 | 6 | ebv-mir-BART17; | tgtatgcctggtgtcccttttagt |
| 600 | + | 139953 | 139975 | 3 | ebv-mir-BART17; | tgtatgccttgtgtccccttagt |
| 601 | + | 139953 | 139976 | 28 | ebv-mir-BART17; | tgtatgcctggtgtccccttagtg |
| 602 | + | 139954 | 139974 | 4 | ebv-mir-BART17; | gtatgcctggtgtccccttag |
| 603 | + | 139954 | 139975 | 3 | ebv-mir-BART17; | gtatgcccggtgtccccttagt |
| 604 | + | 139954 | 139975 | 4 | ebv-mir-BART17; | gtatgcctggtgtccccatagt |
| 605 | + | 139954 | 139975 | 35 | ebv-mir-BART17; | gtatgcctggtgtccccttagt |
| 606 | + | 139955 | 139974 | 4 | ebv-mir-BART17; | tatgcctggtgtccccttag |
| 607 | + | 139955 | 139975 | 5 | ebv-mir-BART17; | tatgcctggtgtccccttagt |
| 608 | + | 139958 | 139975 | 3 | ebv-mir-BART17; | gcctggtgtccccttagt |
| 609 | + | 140033 | 140050 | 26 | ebv-mir-BART6; | taaggttggtccaatcca |
| 610 | + | 140033 | 140051 | 64 | ebv-mir-BART6; | taaggttggtccaatccat |
| 611 | + | 140033 | 140052 | 3 | ebv-mir-BART6; | taaggttggtccaatacata |

| | | | | | | |
|---|---|---|---|---|---|---|
| 612 | + | 140033 | 140052 | 48 | ebv-mir-BART6; | taaggttggtccaatccata |
| 613 | + | 140033 | 140052 | 2 | ebv-mir-BART6; | taaggttggtccaatgcata |
| 614 | + | 140033 | 140052 | 2 | ebv-mir-BART6; | taaggttggtccaattcata |
| 615 | + | 140033 | 140053 | 36 | ebv-mir-BART6; | taaggttggtccaatacatag |
| 616 | + | 140033 | 140053 | 131 | ebv-mir-BART6; | taaggttggtccaatccatag |
| 617 | + | 140033 | 140053 | 3 | ebv-mir-BART6; | taaggttggtccaatgcatag |
| 618 | + | 140033 | 140053 | 41 | ebv-mir-BART6; | taaggttggtccaattcatag |
| 619 | + | 140033 | 140054 | 466 | ebv-mir-BART6; | taaggttggtccaatccatagg |
| 620 | + | 140033 | 140054 | 10 | ebv-mir-BART6; | taaggttggtccaatccctagg |
| 621 | + | 140033 | 140054 | 24 | ebv-mir-BART6; | taaggttggtccaatccgtagg |
| 622 | + | 140033 | 140055 | 3 | ebv-mir-BART6; | taaggttggtccaatccaaaggc |
| 623 | + | 140033 | 140055 | 10 | ebv-mir-BART6; | taaggttggtccaatccagaggc |
| 624 | + | 140033 | 140055 | 212 | ebv-mir-BART6; | taaggttggtccaatccataggc |
| 625 | + | 140033 | 140055 | 25 | ebv-mir-BART6; | taaggttggtccaatccgtaggc |
| 626 | + | 140033 | 140056 | 2 | ebv-mir-BART6; | taaggttggtccaatccataggct |
| 627 | + | 140034 | 140051 | 2 | ebv-mir-BART6; | aaggttggtccaatccat |
| 628 | + | 140034 | 140052 | 2 | ebv-mir-BART6; | aaggttggtccaatccata |
| 629 | + | 140034 | 140053 | 5 | ebv-mir-BART6; | aaggttggtccaatccatag |
| 630 | + | 140059 | 140076 | 3 | - | ttttgggaaaacccgggg |
| 631 | + | 140071 | 140089 | 2 | ebv-mir-BART6; | ccggggatcggactagcct |
| 632 | + | 140071 | 140091 | 2 | ebv-mir-BART6; | ccggggatcggactaccctta |
| 633 | + | 140072 | 140089 | 207 | ebv-mir-BART6; | cggggatcggactagcct |

| | | | | | | |
|---|---|---|---|---|---|---|
| 634 | + | 140072 | 140089 | 3 | ebv-mir-BART6; | cggggatcggactcgcct |
| 635 | + | 140072 | 140090 | 3 | ebv-mir-BART6; | cggggatcggactaacctt |
| 636 | + | 140072 | 140090 | 148 | ebv-mir-BART6; | cggggatcggactagcctt |
| 637 | + | 140072 | 140090 | 47 | ebv-mir-BART6; | cggggatcggactcgcctt |
| 638 | + | 140072 | 140091 | 56 | ebv-mir-BART6; | cggggatcggactaacctta |
| 639 | + | 140072 | 140091 | 6 | ebv-mir-BART6; | cggggatcggactaccctta |
| 640 | + | 140072 | 140091 | 187 | ebv-mir-BART6; | cggggatcggactagcctta |
| 641 | + | 140072 | 140091 | 2 | ebv-mir-BART6; | cggggatcggactagtctta |
| 642 | + | 140072 | 140092 | 22 | ebv-mir-BART6; | cggggatcggactagacttag |
| 643 | + | 140072 | 140092 | 187 | ebv-mir-BART6; | cggggatcggactagccttag |
| 644 | + | 140072 | 140092 | 4 | ebv-mir-BART6; | cggggatcggactagcgttag |
| 645 | + | 140072 | 140092 | 3 | ebv-mir-BART6; | cggggatcggactagctttag |
| 646 | + | 140072 | 140092 | 6 | ebv-mir-BART6; | cggggatcggactagtcttag |
| 647 | + | 140072 | 140093 | 19 | ebv-mir-BART6; | cggggatcggactagcattaga |
| 648 | + | 140072 | 140093 | 40 | ebv-mir-BART6; | cggggatcggactagccataga |
| 649 | + | 140072 | 140093 | 20 | ebv-mir-BART6; | cggggatcggactagccctaga |
| 650 | + | 140072 | 140093 | 8 | ebv-mir-BART6; | cggggatcggactagccgtaga |
| 651 | + | 140072 | 140093 | 377 | ebv-mir-BART6; | cggggatcggactagccttaga |
| 652 | + | 140072 | 140093 | 21 | ebv-mir-BART6; | cggggatcggactagcgttaga |
| 653 | + | 140072 | 140093 | 3 | ebv-mir-BART6; | cggggatcggactagctttaga |
| 654 | + | 140072 | 140094 | 2 | ebv-mir-BART6; | cggggatcggactagccttagag |
| 655 | + | 140072 | 140095 | 4 | ebv-mir-BART6; | cggggatcggactagccttagagt |

| | | | | | | |
|---|---|---|---|---|---|---|
| 656 | + | 140072 | 140096 | 2 | ebv-mir-BART6; | cggggatcggactagcctaagagta |
| 657 | + | 144282 | 144299 | 3 | - | atccataagccccgcctt |
| 658 | + | 144559 | 144576 | 8 | - | gcccttggcccaaccccc |
| 659 | + | 144565 | 144583 | 4 | - | ggcccgacccccgccttgg |
| 660 | + | 144566 | 144583 | 3 | - | gccccacccccgccttgg |
| 661 | + | 144566 | 144583 | 2 | - | gcccgacccccgccttgg |
| 662 | + | 144566 | 144583 | 9 | - | gccctacccccgccttgg |
| 663 | + | 145514 | 145531 | 8 | ebv-mir-BART21; | tcactagtgaaggcaact |
| 664 | + | 145514 | 145532 | 18 | ebv-mir-BART21; | tcactagtgaaggcaacta |
| 665 | + | 145514 | 145533 | 9 | ebv-mir-BART21; | tcactagtgaaggcaactaa |
| 666 | + | 145514 | 145534 | 48 | ebv-mir-BART21; | tcactagtgaaggcaactaac |
| 667 | + | 145514 | 145534 | 2 | ebv-mir-BART21; | tcactagtgaaggcacctaac |
| 668 | + | 145514 | 145534 | 2 | ebv-mir-BART21; | tcactagtgaaggctactaac |
| 669 | + | 145514 | 145535 | 2 | ebv-mir-BART21; | tcactagtgaaggcaaccaaca |
| 670 | + | 145514 | 145535 | 13 | ebv-mir-BART21; | tcactagtgaaggcaactaaca |
| 671 | + | 145514 | 145536 | 2 | ebv-mir-BART21; | tcactagtgaaggcaacaaacac |
| 672 | + | 145514 | 145536 | 12 | ebv-mir-BART21; | tcactagtgaaggcaaccaacac |
| 673 | + | 145514 | 145536 | 4 | ebv-mir-BART21; | tcactagtgaaggcaacgaacac |
| 674 | + | 145514 | 145536 | 56 | ebv-mir-BART21; | tcactagtgaaggcaactaacac |
| 675 | + | 145548 | 145566 | 8 | ebv-mir-BART21; | ctagttgtgcccactggtg |
| 676 | + | 145548 | 145569 | 4 | ebv-mir-BART21; | ctagttgtgcccactggtgttt |
| 677 | + | 145943 | 145961 | 3 | - | gaaagacgggtgtcctggc |

| | | | | | | |
|---|---|---|---|---|---|---|
| 678 | + | 145962 | 145979 | 251 | ebv-mir-BART18; | tcaagttcgcacttccta |
| 679 | + | 145962 | 145980 | 8 | ebv-mir-BART18; | tcaagttcgcactacctat |
| 680 | + | 145962 | 145980 | 3 | ebv-mir-BART18; | tcaagttcgcactccctat |
| 681 | + | 145962 | 145980 | 60 | ebv-mir-BART18; | tcaagttcgcacttcctat |
| 682 | + | 145962 | 145981 | 96 | ebv-mir-BART18; | tcaagttcgcacttcctata |
| 683 | + | 145962 | 145981 | 2 | ebv-mir-BART18; | tcaagttcgcacttcgtata |
| 684 | + | 145962 | 145982 | 26 | ebv-mir-BART18; | tcaagttcgcacttcatatac |
| 685 | + | 145962 | 145982 | 6 | ebv-mir-BART18; | tcaagttcgcacttccaatac |
| 686 | + | 145962 | 145982 | 18 | ebv-mir-BART18; | tcaagttcgcacttcccatac |
| 687 | + | 145962 | 145982 | 4 | ebv-mir-BART18; | tcaagttcgcacttccgatac |
| 688 | + | 145962 | 145982 | 490 | ebv-mir-BART18; | tcaagttcgcacttcctatac |
| 689 | + | 145962 | 145982 | 13 | ebv-mir-BART18; | tcaagttcgcacttcgtatac |
| 690 | + | 145962 | 145982 | 4 | ebv-mir-BART18; | tcaagttcgcacttcttatac |
| 691 | + | 145962 | 145983 | 87 | ebv-mir-BART18; | tcaagttcgcacttccaataca |
| 692 | + | 145962 | 145983 | 45 | ebv-mir-BART18; | tcaagttcgcacttcccataca |
| 693 | + | 145962 | 145983 | 111 | ebv-mir-BART18; | tcaagttcgcacttccgataca |
| 694 | + | 145962 | 145983 | 279 | ebv-mir-BART18; | tcaagttcgcacttcctataca |
| 695 | + | 145962 | 145983 | 6 | ebv-mir-BART18; | tcaagttcgcacttcctctaca |
| 696 | + | 145962 | 145983 | 6 | ebv-mir-BART18; | tcaagttcgcacttcctgtaca |
| 697 | + | 145962 | 145983 | 2 | ebv-mir-BART18; | tcaagttcgcacttcctttaca |
| 698 | + | 145962 | 145984 | 22 | ebv-mir-BART18; | tcaagttcgcacttcctatacag |
| 699 | + | 145962 | 145985 | 6 | ebv-mir-BART18; | tcaagttcgcacttcctatacagt |

| | | | | | | |
|---|---|---|---|---|---|---|
| 700 | + | 145963 | 145981 | 11 | ebv-mir-BART18; | caagttcgcacttcctata |
| 701 | + | 145963 | 145983 | 2 | ebv-mir-BART18; | caagttcgcacttccaataca |
| 702 | + | 145963 | 145983 | 13 | ebv-mir-BART18; | caagttcgcacttccgataca |
| 703 | + | 145963 | 145983 | 22 | ebv-mir-BART18; | caagttcgcacttcctataca |
| 704 | + | 145998 | 146015 | 2 | ebv-mir-BART18; | tatcggaagtttgggctt |
| 705 | + | 145998 | 146016 | 3 | ebv-mir-BART18; | tatcggaagtttgggcttc |
| 706 | + | 145998 | 146017 | 12 | ebv-mir-BART18; | tatcggaagtttgggcttcg |
| 707 | + | 145998 | 146018 | 2 | ebv-mir-BART18; | tatcggaagtttgggcatcgt |
| 708 | + | 145998 | 146019 | 4 | ebv-mir-BART18; | tatcggaagtttgggattcgtc |
| 709 | + | 145998 | 146019 | 2 | ebv-mir-BART18; | tatcggaagtttgggctccgtc |
| 710 | + | 145998 | 146019 | 2 | ebv-mir-BART18; | tatcggaagtttgggctgcgtc |
| 711 | + | 145998 | 146019 | 9 | ebv-mir-BART18; | tatcggaagtttgggcttcgtc |
| 712 | + | 145998 | 146020 | 4 | ebv-mir-BART18; | tatcggaagtttgggcttcgtcc |
| 713 | + | 145999 | 146017 | 2 | ebv-mir-BART18; | atcggaagtttgggcttcg |
| 714 | + | 146438 | 146459 | 2 | ebv-mir-BART7; | tcctggaccttgactatgaaac |
| 715 | + | 146439 | 146456 | 7 | ebv-mir-BART7; | cctggaccttgactatga |
| 716 | + | 146439 | 146457 | 12 | ebv-mir-BART7; | cctggaccttgactatgaa |
| 717 | + | 146439 | 146458 | 4 | ebv-mir-BART7; | cctggaccttgactatgaaa |
| 718 | + | 146439 | 146459 | 15 | ebv-mir-BART7; | cctggaccttgactaagaaac |
| 719 | + | 146439 | 146459 | 5 | ebv-mir-BART7; | cctggaccttgactacgaaac |
| 720 | + | 146439 | 146459 | 4 | ebv-mir-BART7; | cctggaccttgactaggaaac |
| 721 | + | 146439 | 146459 | 2 | ebv-mir-BART7; | cctggaccttgactataaaac |

| | | | | | | |
|---|---|---|---|---|---|---|
| 722 | + | 146439 | 146459 | 58 | ebv-mir-BART7; | cctggaccttgactatgaaac |
| 723 | + | 146439 | 146460 | 20 | ebv-mir-BART7; | cctggaccttgactataaaaca |
| 724 | + | 146439 | 146460 | 95 | ebv-mir-BART7; | cctggaccttgactatgaaaca |
| 725 | + | 146439 | 146460 | 2 | ebv-mir-BART7; | cctggaccttgactatgcaaca |
| 726 | + | 146439 | 146460 | 3 | ebv-mir-BART7; | cctggaccttgactatggaaca |
| 727 | + | 146439 | 146461 | 9 | ebv-mir-BART7; | cctggaccttgactatgaaacaa |
| 728 | + | 146475 | 146492 | 9 | ebv-mir-BART7; | catcatagtccaatgtcc |
| 729 | + | 146475 | 146492 | 3 | ebv-mir-BART7; | catcatagtccagggtcc |
| 730 | + | 146475 | 146492 | 878 | ebv-mir-BART7; | catcatagtccagtgtcc |
| 731 | + | 146475 | 146492 | 3 | ebv-mir-BART7; | catcatagtccggtgtcc |
| 732 | + | 146475 | 146493 | 11 | ebv-mir-BART7; | catcatagtccagagtcca |
| 733 | + | 146475 | 146493 | 4 | ebv-mir-BART7; | catcatagtccagcgtcca |
| 734 | + | 146475 | 146493 | 13 | ebv-mir-BART7; | catcatagtccagggtcca |
| 735 | + | 146475 | 146493 | 36 | ebv-mir-BART7; | catcatagtccagtatcca |
| 736 | + | 146475 | 146493 | 2 | ebv-mir-BART7; | catcatagtccagtctcca |
| 737 | + | 146475 | 146493 | 1005 | ebv-mir-BART7; | catcatagtccagtgtcca |
| 738 | + | 146475 | 146493 | 2 | ebv-mir-BART7; | catcatagtccggtgtcca |
| 739 | + | 146475 | 146494 | 92 | ebv-mir-BART7; | catcatagtccagtatccag |
| 740 | + | 146475 | 146494 | 4 | ebv-mir-BART7; | catcatagtccagtctccag |
| 741 | + | 146475 | 146494 | 16 | ebv-mir-BART7; | catcatagtccagtgaccag |
| 742 | + | 146475 | 146494 | 34 | ebv-mir-BART7; | catcatagtccagtgcccag |
| 743 | + | 146475 | 146494 | 5 | ebv-mir-BART7; | catcatagtccagtggccag |

| | | | | | | |
|---|---|---|---|---|---|---|
| 744 | + | 146475 | 146494 | 1418 | ebv-mir-BART7; | catcatagtccagtgtccag |
| 745 | + | 146475 | 146495 | 2 | ebv-mir-BART7; | catcatagtccagtatccagg |
| 746 | + | 146475 | 146495 | 2 | ebv-mir-BART7; | catcatagtccagtgcccagg |
| 747 | + | 146475 | 146495 | 6 | ebv-mir-BART7; | catcatagtccagtgtacagg |
| 748 | + | 146475 | 146495 | 1114 | ebv-mir-BART7; | catcatagtccagtgtccagg |
| 749 | + | 146475 | 146495 | 2 | ebv-mir-BART7; | catcatagtccggtgtccagg |
| 750 | + | 146475 | 146496 | 14 | ebv-mir-BART7; | catcatagtccagtgtcaaggg |
| 751 | + | 146475 | 146496 | 1010 | ebv-mir-BART7; | catcatagtccagtgtccaggg |
| 752 | + | 146475 | 146496 | 25 | ebv-mir-BART7; | catcatagtccagtgtcgaggg |
| 753 | + | 146475 | 146496 | 93 | ebv-mir-BART7; | catcatagtccagtgtctaggg |
| 754 | + | 146475 | 146496 | 12 | ebv-mir-BART7; | catcatagtccagtgttcaggg |
| 755 | + | 146475 | 146497 | 1287 | ebv-mir-BART7; | catcatagtccagtgtccaggga |
| 756 | + | 146475 | 146497 | 12 | ebv-mir-BART7; | catcatagtccagtgtcccggga |
| 757 | + | 146475 | 146497 | 105 | ebv-mir-BART7; | catcatagtccagtgtccgggga |
| 758 | + | 146475 | 146497 | 27 | ebv-mir-BART7; | catcatagtccagtgtctaggga |
| 759 | + | 146475 | 146498 | 24 | ebv-mir-BART7; | catcatagtccagtgtcaagggac |
| 760 | + | 146475 | 146498 | 16 | ebv-mir-BART7; | catcatagtccagtgtccaaggac |
| 761 | + | 146475 | 146498 | 6 | ebv-mir-BART7; | catcatagtccagtgtccacggac |
| 762 | + | 146475 | 146498 | 546 | ebv-mir-BART7; | catcatagtccagtgtccagggac |
| 763 | + | 146475 | 146498 | 11 | ebv-mir-BART7; | catcatagtccagtgtccatggac |
| 764 | + | 146475 | 146498 | 3 | ebv-mir-BART7; | catcatagtccagtgtcccgggac |
| 765 | + | 146475 | 146498 | 31 | ebv-mir-BART7; | catcatagtccagtgtccggggac |

| | | | | | | |
|---|---|---|---|---|---|---|
| 766 | + | 146475 | 146498 | 6 | ebv-mir-BART7; | catcatagtccagtgtcctgggac |
| 767 | + | 146475 | 146498 | 14 | ebv-mir-BART7; | catcatagtccagtgtcgagggac |
| 768 | + | 146475 | 146498 | 25 | ebv-mir-BART7; | catcatagtccagtgtctagggac |
| 769 | + | 146475 | 146498 | 2 | ebv-mir-BART7; | catcatagtctagtgtccagggac |
| 770 | + | 146475 | 146499 | 24 | ebv-mir-BART7; | catcatagtccagtgtccagggaca |
| 771 | + | 146476 | 146495 | 2 | ebv-mir-BART7; | atcatagtccagtgtccagg |
| 772 | + | 146476 | 146497 | 11 | ebv-mir-BART7; | atcatagtccagtgtccaggga |
| 773 | + | 146772 | 146789 | 3 | ebv-mir-BART8; | tacggtttcctaaattgt |
| 774 | + | 146772 | 146789 | 3 | ebv-mir-BART8; | tacggtttcctacattgt |
| 775 | + | 146772 | 146789 | 68 | ebv-mir-BART8; | tacggtttcctagattgt |
| 776 | + | 146772 | 146790 | 47 | ebv-mir-BART8; | tacggtttcctagattgta |
| 777 | + | 146772 | 146791 | 8 | ebv-mir-BART8; | tacggtttcctagaatgtac |
| 778 | + | 146772 | 146791 | 5 | ebv-mir-BART8; | tacggtttcctagactgtac |
| 779 | + | 146772 | 146791 | 3 | ebv-mir-BART8; | tacggtttcctagagtgtac |
| 780 | + | 146772 | 146791 | 5 | ebv-mir-BART8; | tacggtttcctagatagtac |
| 781 | + | 146772 | 146791 | 2 | ebv-mir-BART8; | tacggtttcctagatcgtac |
| 782 | + | 146772 | 146791 | 23 | ebv-mir-BART8; | tacggtttcctagatggtac |
| 783 | + | 146772 | 146791 | 124 | ebv-mir-BART8; | tacggtttcctagattgtac |
| 784 | + | 146772 | 146792 | 55 | ebv-mir-BART8; | tacggtttcctagatagtaca |
| 785 | + | 146772 | 146792 | 18 | ebv-mir-BART8; | tacggtttcctagatcgtaca |
| 786 | + | 146772 | 146792 | 13 | ebv-mir-BART8; | tacggtttcctagatggtaca |
| 787 | + | 146772 | 146792 | 10 | ebv-mir-BART8; | tacggtttcctagattataca |

| | | | | | | |
|---|---|---|---|---|---|---|
| 788 | + | 146772 | 146792 | 2 | ebv-mir-BART8; | tacggtttcctagattctaca |
| 789 | + | 146772 | 146792 | 193 | ebv-mir-BART8; | tacggtttcctagattgtaca |
| 790 | + | 146772 | 146793 | 2 | ebv-mir-BART8; | tacggcttcctagattgtacag |
| 791 | + | 146772 | 146793 | 85 | ebv-mir-BART8; | tacggtttcctagattatacag |
| 792 | + | 146772 | 146793 | 5 | ebv-mir-BART8; | tacggtttcctagattgcacag |
| 793 | + | 146772 | 146793 | 16 | ebv-mir-BART8; | tacggtttcctagattggacag |
| 794 | + | 146772 | 146793 | 1374 | ebv-mir-BART8; | tacggtttcctagattgtacag |
| 795 | + | 146772 | 146794 | 24 | ebv-mir-BART8; | tacggtttcctagattgtacaga |
| 796 | + | 146773 | 146793 | 16 | ebv-mir-BART8; | acggtttcctagattgtacag |
| 797 | + | 146806 | 146830 | 2 | ebv-mir-BART8; | tgtcacaatctatggggtcgtagac |
| 798 | + | 146807 | 146824 | 2 | ebv-mir-BART8; | gtcaaaatctatggggtc |
| 799 | + | 146807 | 146824 | 3 | ebv-mir-BART8; | gtcacaatctatgaggtc |
| 800 | + | 146807 | 146825 | 7 | ebv-mir-BART8; | gtcacaatctacggggtcg |
| 801 | + | 146807 | 146825 | 9 | ebv-mir-BART8; | gtcacaatctatagggtcg |
| 802 | + | 146807 | 146825 | 13 | ebv-mir-BART8; | gtcacaatctatgaggtcg |
| 803 | + | 146807 | 146825 | 8 | ebv-mir-BART8; | gtcacaatctatggagtcg |
| 804 | + | 146807 | 146825 | 2 | ebv-mir-BART8; | gtcactatctatggggtcg |
| 805 | + | 146807 | 146826 | 5 | ebv-mir-BART8; | gtcacaatctatgggatcgt |
| 806 | + | 146807 | 146827 | 26 | ebv-mir-BART8; | gtcacaatctatggggacgta |
| 807 | + | 146807 | 146827 | 27 | ebv-mir-BART8; | gtcacaatctatggggccgta |
| 808 | + | 146807 | 146827 | 7 | ebv-mir-BART8; | gtcacaatctatgggggcgta |
| 809 | + | 146807 | 146828 | 14 | ebv-mir-BART8; | gtcacaatctatggggacgtag |

| | | | | | | |
|---|---|---|---|---|---|---|
| 810 | + | 146807 | 146828 | 20 | ebv-mir-BART8; | gtcacaatctatggggtagtag |
| 811 | + | 146807 | 146828 | 18 | ebv-mir-BART8; | gtcacaatctatggggttgtag |
| 812 | + | 146807 | 146829 | 3 | ebv-mir-BART8; | gtcacaatctatggggtagtaga |
| 813 | + | 146807 | 146830 | 2 | ebv-mir-BART8; | gtcacaatctat-gggtcgtagac |
| 814 | + | 146807 | 146830 | 178 | ebv-mir-BART8; | gtcacaatctatggggtagtagac |
| 815 | + | 146807 | 146830 | 13 | ebv-mir-BART8; | gtcacaatctatggggtggtagac |
| 816 | + | 146807 | 146830 | 88 | ebv-mir-BART8; | gtcacaatctatggggttgtagac |
| 817 | + | 146807 | 146830 | 3 | ebv-mir-BART8; | gtcataatctatggggtcgtagac |
| 818 | + | 146807 | 146831 | 8 | ebv-mir-BART8; | gtcacaatctatggggtagtagaca |
| 819 | + | 146808 | 146825 | 6 | ebv-mir-BART8; | tcacaatctatggggtcg |
| 820 | + | 146808 | 146827 | 2 | ebv-mir-BART8; | tcacaatctatggggtcgta |
| 821 | + | 146808 | 146828 | 5 | ebv-mir-BART8; | tcacaatctatggggtcgtag |
| 822 | + | 146808 | 146829 | 2 | ebv-mir-BART8; | tcacaatctatggggtcgtaga |
| 823 | + | 146808 | 146830 | 3 | ebv-mir-BART8; | tcacaatctatggggtcatagac |
| 824 | + | 146808 | 146830 | 11 | ebv-mir-BART8; | tcacaatctatggggtcgtagac |
| 825 | + | 146808 | 146830 | 4 | ebv-mir-BART8; | tcacaatctatggggttgtagac |
| 826 | + | 146919 | 146943 | 2 | - | gggctttgtgatagctaatgaatgt |
| 827 | + | 146959 | 146976 | 5 | ebv-mir-BART9; | tactggaccctgaattgg |
| 828 | + | 146959 | 146977 | 13 | ebv-mir-BART9; | tactggaccctgaattgga |
| 829 | + | 146959 | 146978 | 4 | ebv-mir-BART9; | tactggaccctgaataggaa |
| 830 | + | 146959 | 146978 | 9 | ebv-mir-BART9; | tactggaccctgaattggaa |
| 831 | + | 146959 | 146979 | 15 | ebv-mir-BART9; | tactggaccctgaattggaaa |

| | | | | | | |
|---|---|---|---|---|---|---|
| 832 | + | 146959 | 146980 | 3 | ebv-mir-BART9; | tactggaccctgaataggaaac |
| 833 | + | 146959 | 146980 | 3 | ebv-mir-BART9; | tactggaccctgaatgggaaac |
| 834 | + | 146959 | 146980 | 41 | ebv-mir-BART9; | tactggaccctgaattggaaac |
| 835 | + | 146959 | 146981 | 21 | ebv-mir-BART9; | tactggaccctgaattgaaaaca |
| 836 | + | 146959 | 146981 | 23 | ebv-mir-BART9; | tactggaccctgaattggaaaca |
| 837 | + | 146959 | 146981 | 4 | ebv-mir-BART9; | tactggaccctgaattgggaaca |
| 838 | + | 146959 | 146982 | 3 | ebv-mir-BART9; | tactggaccctgaattggaaacag |
| 839 | + | 146959 | 146982 | 5 | ebv-mir-BART9; | tactggaccctgaattggagacag |
| 840 | + | 146960 | 146982 | 2 | ebv-mir-BART9; | actggaccctgaattggaaacag |
| 841 | + | 146996 | 147013 | 24 | ebv-mir-BART9; | gtaacacttcatgggtcc |
| 842 | + | 146996 | 147014 | 63 | ebv-mir-BART9; | gtaacacttcatgggtccc |
| 843 | + | 146996 | 147015 | 2 | ebv-mir-BART9; | gtaacacttcatgggccccg |
| 844 | + | 146996 | 147015 | 93 | ebv-mir-BART9; | gtaacacttcatgggtcccg |
| 845 | + | 146996 | 147016 | 2 | ebv-mir-BART9; | gtaacacttcatggctcccgt |
| 846 | + | 146996 | 147016 | 6 | ebv-mir-BART9; | gtaacacttcatgggacccgt |
| 847 | + | 146996 | 147016 | 11 | ebv-mir-BART9; | gtaacacttcatgggccccgt |
| 848 | + | 146996 | 147016 | 35 | ebv-mir-BART9; | gtaacacttcatgggtcccgt |
| 849 | + | 146996 | 147017 | 3 | ebv-mir-BART9; | gtaacacttcatgggtaccgta |
| 850 | + | 146996 | 147017 | 2 | ebv-mir-BART9; | gtaacacttcatgggtcacgta |
| 851 | + | 146996 | 147017 | 113 | ebv-mir-BART9; | gtaacacttcatgggtcccgta |
| 852 | + | 146996 | 147017 | 8 | ebv-mir-BART9; | gtaacacttcatgggtcgcgta |
| 853 | + | 146996 | 147017 | 2 | ebv-mir-BART9; | gtaacacttcatgggtctcgta |

| | | | | | | |
|---|---|---|---|---|---|---|
| 854 | + | 146996 | 147017 | 3 | ebv-mir-BART9; | gtaacacttcatgggtgccgta |
| 855 | + | 146996 | 147018 | 2 | ebv-mir-BART9; | gtaacacttcatgggtcacgtag |
| 856 | + | 146996 | 147018 | 80 | ebv-mir-BART9; | gtaacacttcatgggtcccgtag |
| 857 | + | 146996 | 147018 | 2 | ebv-mir-BART9; | gtaacacttcatgggtcctgtag |
| 858 | + | 146996 | 147018 | 5 | ebv-mir-BART9; | gtaacacttcatgggtcgcgtag |
| 859 | + | 146996 | 147018 | 7 | ebv-mir-BART9; | gtaacacttcatgggtctcgtag |
| 860 | + | 146996 | 147019 | 65 | ebv-mir-BART9; | gtaacacttcatgggtcccgtagt |
| 861 | + | 146996 | 147020 | 4 | ebv-mir-BART9; | gtaacacttcatgggtcccgtagtg |
| 862 | + | 146997 | 147014 | 13 | ebv-mir-BART9; | taacacttcatgagtccc |
| 863 | + | 146997 | 147014 | 6 | ebv-mir-BART9; | taacacttcatggatccc |
| 864 | + | 146997 | 147014 | 1592 | ebv-mir-BART9; | taacacttcatgggtccc |
| 865 | + | 146997 | 147014 | 4 | ebv-mir-BART9; | taacacttcattggtccc |
| 866 | + | 146997 | 147015 | 8 | ebv-mir-BART9; | taacactccatgggtcccg |
| 867 | + | 146997 | 147015 | 38 | ebv-mir-BART9; | taacacttcataggtcccg |
| 868 | + | 146997 | 147015 | 46 | ebv-mir-BART9; | taacacttcatgagtcccg |
| 869 | + | 146997 | 147015 | 69 | ebv-mir-BART9; | taacacttcatggatcccg |
| 870 | + | 146997 | 147015 | 79 | ebv-mir-BART9; | taacacttcatgggccccg |
| 871 | + | 146997 | 147015 | 7 | ebv-mir-BART9; | taacacttcatggggcccg |
| 872 | + | 146997 | 147015 | 2976 | ebv-mir-BART9; | taacacttcatgggtcccg |
| 873 | + | 146997 | 147016 | 9 | ebv-mir-BART9; | taacacttcatgggccccgt |
| 874 | + | 146997 | 147016 | 21 | ebv-mir-BART9; | taacacttcatggggcccgt |
| 875 | + | 146997 | 147016 | 5 | ebv-mir-BART9; | taacacttcatgggtaccgt |

| | | | | | | |
|---|---|---|---|---|---|---|
| 876 | + | 146997 | 147016 | 708 | ebv-mir-BART9; | taacacttcatgggtcccgt |
| 877 | + | 146997 | 147016 | 3 | ebv-mir-BART9; | taacccttcatgggtcccgt |
| 878 | + | 146997 | 147017 | 2 | ebv-mir-BART9; | taacacctcatgggtcccgta |
| 879 | + | 146997 | 147017 | 2 | ebv-mir-BART9; | taacacttcatggctcccgta |
| 880 | + | 146997 | 147017 | 13 | ebv-mir-BART9; | taacacttcatgggtaccgta |
| 881 | + | 146997 | 147017 | 45 | ebv-mir-BART9; | taacacttcatgggtcacgta |
| 882 | + | 146997 | 147017 | 1600 | ebv-mir-BART9; | taacacttcatgggtcccgta |
| 883 | + | 146997 | 147017 | 19 | ebv-mir-BART9; | taacacttcatgggtcgcgta |
| 884 | + | 146997 | 147017 | 25 | ebv-mir-BART9; | taacacttcatgggtctcgta |
| 885 | + | 146997 | 147018 | 88 | ebv-mir-BART9; | taacacttcatgggtcacgtag |
| 886 | + | 146997 | 147018 | 60 | ebv-mir-BART9; | taacacttcatgggtccagtag |
| 887 | + | 146997 | 147018 | 2284 | ebv-mir-BART9; | taacacttcatgggtcccgtag |
| 888 | + | 146997 | 147018 | 80 | ebv-mir-BART9; | taacacttcatgggtccggtag |
| 889 | + | 146997 | 147018 | 21 | ebv-mir-BART9; | taacacttcatgggtcctgtag |
| 890 | + | 146997 | 147018 | 15 | ebv-mir-BART9; | taacacttcatgggtcgcgtag |
| 891 | + | 146997 | 147018 | 101 | ebv-mir-BART9; | taacacttcatgggtctcgtag |
| 892 | + | 146997 | 147018 | 3 | ebv-mir-BART9; | taacacttcatgggttccgtag |
| 893 | + | 146997 | 147019 | 5 | ebv-mir-BART9; | taacacctcatgggtcccgtagt |
| 894 | + | 146997 | 147019 | 17 | ebv-mir-BART9; | taacacttcatggatcccgtagt |
| 895 | + | 146997 | 147019 | 3 | ebv-mir-BART9; | taacacttcatgggccccgtagt |
| 896 | + | 146997 | 147019 | 322 | ebv-mir-BART9; | taacacttcatgggtccagtagt |
| 897 | + | 146997 | 147019 | 82 | ebv-mir-BART9; | taacacttcatgggtcccatagt |

| | | | | | | |
|---|---|---|---|---|---|---|
| 898 | + | 146997 | 147019 | 15 | ebv-mir-BART9; | taacacttcatgggtcccctagt |
| 899 | + | 146997 | 147019 | 5096 | ebv-mir-BART9; | taacacttcatgggtcccgtagt |
| 900 | + | 146997 | 147019 | 45 | ebv-mir-BART9; | taacacttcatgggtcccttagt |
| 901 | + | 146997 | 147019 | 149 | ebv-mir-BART9; | taacacttcatgggtccggtagt |
| 902 | + | 146997 | 147019 | 197 | ebv-mir-BART9; | taacacttcatgggtcctgtagt |
| 903 | + | 146997 | 147019 | 4 | ebv-mir-BART9; | taacacttcatgggtctcgtagt |
| 904 | + | 146997 | 147019 | 5 | ebv-mir-BART9; | taacacttcgtgggtcccgtagt |
| 905 | + | 146997 | 147019 | 7 | ebv-mir-BART9; | taacactttatgggtcccgtagt |
| 906 | + | 146997 | 147019 | 4 | ebv-mir-BART9; | taacatttcatgggtcccgtagt |
| 907 | + | 146997 | 147019 | 2 | ebv-mir-BART9; | taacgcttcatgggtcccgtagt |
| 908 | + | 146997 | 147020 | 4 | ebv-mir-BART9; | taacacttcatgggtcccgcagtg |
| 909 | + | 146997 | 147020 | 95 | ebv-mir-BART9; | taacacttcatgggtcccgtagtg |
| 910 | + | 146997 | 147020 | 3 | ebv-mir-BART9; | taacacttcatgggtccggtagtg |
| 911 | + | 146997 | 147021 | 11 | ebv-mir-BART9; | taacacttcatgggtcccgtagtga |
| 912 | + | 146998 | 147015 | 2 | ebv-mir-BART9; | aacacttcatggatcccg |
| 913 | + | 146998 | 147015 | 3 | ebv-mir-BART9; | aacacttcatgggccccg |
| 914 | + | 146998 | 147015 | 91 | ebv-mir-BART9; | aacacttcatgggtcccg |
| 915 | + | 146998 | 147016 | 17 | ebv-mir-BART9; | aacacttcatgggtcccgt |
| 916 | + | 146998 | 147017 | 3 | ebv-mir-BART9; | aacacttcatgggtcacgta |
| 917 | + | 146998 | 147017 | 34 | ebv-mir-BART9; | aacacttcatgggtcccgta |
| 918 | + | 146998 | 147018 | 2 | ebv-mir-BART9; | aacacttcatgggtccagtag |
| 919 | + | 146998 | 147018 | 79 | ebv-mir-BART9; | aacacttcatgggtcccgtag |

| | | | | | | |
|---|---|---|---|---|---|---|
| 920 | + | 146998 | 147019 | 43 | ebv-mir-BART9; | aacacttcatgggtccagtagt |
| 921 | + | 146998 | 147019 | 179 | ebv-mir-BART9; | aacacttcatgggtcccgtagt |
| 922 | + | 146998 | 147019 | 12 | ebv-mir-BART9; | aacacttcatgggtcctgtagt |
| 923 | + | 146998 | 147020 | 2 | ebv-mir-BART9; | aacacttcatgggtcccgtagtg |
| 924 | + | 147002 | 147019 | 7 | ebv-mir-BART9; | cttcatgggtcccgtagt |
| 925 | + | 147169 | 147190 | 7 | ebv-mir-BART22; | tgctagaccctggagttgaacc |
| 926 | + | 147203 | 147220 | 25 | ebv-mir-BART22; | ttacaaagtcatggtcta |
| 927 | + | 147203 | 147221 | 51 | ebv-mir-BART22; | ttacaaagtcatggtctag |
| 928 | + | 147203 | 147222 | 29 | ebv-mir-BART22; | ttacaaagtcatggtctagt |
| 929 | + | 147203 | 147223 | 2 | ebv-mir-BART22; | ttacaaagtcatggcctagta |
| 930 | + | 147203 | 147223 | 26 | ebv-mir-BART22; | ttacaaagtcatggtctagta |
| 931 | + | 147203 | 147223 | 2 | ebv-mir-BART22; | ttacaaagtcatggtttagta |
| 932 | + | 147203 | 147224 | 2 | ebv-mir-BART22; | ttacaaagtcatggtcaagtag |
| 933 | + | 147203 | 147224 | 20 | ebv-mir-BART22; | ttacaaagtcatggtcgagtag |
| 934 | + | 147203 | 147224 | 102 | ebv-mir-BART22; | ttacaaagtcatggtctagtag |
| 935 | + | 147203 | 147224 | 3 | ebv-mir-BART22; | ttacaaagtcatggtctggtag |
| 936 | + | 147203 | 147225 | 2 | ebv-mir-BART22; | ttacaaagtcatggtccagtagt |
| 937 | + | 147203 | 147225 | 4 | ebv-mir-BART22; | ttacaaagtcatggtctaatagt |
| 938 | + | 147203 | 147225 | 154 | ebv-mir-BART22; | ttacaaagtcatggtctagtagt |
| 939 | + | 147203 | 147225 | 2 | ebv-mir-BART22; | ttacaaagtcatggtctattagt |
| 940 | + | 147203 | 147225 | 3 | ebv-mir-BART22; | ttacaaagtcatggtctcgtagt |
| 941 | + | 147203 | 147225 | 10 | ebv-mir-BART22; | ttacaaagtcatggtctggtagt |

| | | | | | | |
|---|---|---|---|---|---|---|
| 942 | + | 147204 | 147221 | 2 | ebv-mir-BART22; | tacaaagtcatggtctag |
| 943 | + | 147204 | 147223 | 3 | ebv-mir-BART22; | tacaaagtcatggtctagta |
| 944 | + | 147204 | 147224 | 2 | ebv-mir-BART22; | tacaaagtcatggtcgagtag |
| 945 | + | 147204 | 147224 | 8 | ebv-mir-BART22; | tacaaagtcatggtctagtag |
| 946 | + | 147204 | 147225 | 4 | ebv-mir-BART22; | tacaaagtcatggtctaatagt |
| 947 | + | 147204 | 147225 | 42 | ebv-mir-BART22; | tacaaagtcatggtctagtagt |
| 948 | + | 147204 | 147225 | 2 | ebv-mir-BART22; | tacaaagtcatggtctggtagt |
| 949 | + | 147321 | 147340 | 9 | ebv-mir-BART10; | gccacctctttggttctgta |
| 950 | + | 147321 | 147341 | 3 | ebv-mir-BART10; | gccacctctttggttatgtac |
| 951 | + | 147321 | 147341 | 35 | ebv-mir-BART10; | gccacctctttggttccgtac |
| 952 | + | 147321 | 147341 | 57 | ebv-mir-BART10; | gccacctctttggttctgtac |
| 953 | + | 147321 | 147344 | 2 | ebv-mir-BART10; | gccacctctttggttctgtacata |
| 954 | + | 147356 | 147373 | 378 | ebv-mir-BART10; | tacataaccatggagttg |
| 955 | + | 147356 | 147374 | 50 | ebv-mir-BART10; | tacataaccatggagttgg |
| 956 | + | 147356 | 147375 | 3 | ebv-mir-BART10; | tacataaccatggaattggc |
| 957 | + | 147356 | 147375 | 3 | ebv-mir-BART10; | tacataaccatggagatggc |
| 958 | + | 147356 | 147375 | 364 | ebv-mir-BART10; | tacataaccatggagttggc |
| 959 | + | 147356 | 147376 | 68 | ebv-mir-BART10; | tacataaccatggagttggct |
| 960 | + | 147356 | 147377 | 49 | ebv-mir-BART10; | tacataaccatggagttagctg |
| 961 | + | 147356 | 147377 | 172 | ebv-mir-BART10; | tacataaccatggagttggctg |
| 962 | + | 147356 | 147378 | 68 | ebv-mir-BART10; | tacataaccatggagttagctgt |
| 963 | + | 147356 | 147378 | 418 | ebv-mir-BART10; | tacataaccatggagttgactgt |

| | | | | | | |
|---|---|---|---|---|---|---|
| 964 | + | 147356 | 147378 | 11 | ebv-mir-BART10; | tacataaccatggagttgcctgt |
| 965 | + | 147356 | 147378 | 163 | ebv-mir-BART10; | tacataaccatggagttggctgt |
| 966 | + | 147356 | 147378 | 10 | ebv-mir-BART10; | tacataaccatggagttgtctgt |
| 967 | + | 147356 | 147378 | 6 | ebv-mir-BART10; | tacataaccatggagtttgctgt |
| 968 | + | 147356 | 147378 | 11 | ebv-mir-BART10; | tacataaccgtggagttggctgt |
| 969 | + | 147356 | 147379 | 4 | ebv-mir-BART10; | tacataaccatggagttgactgtg |
| 970 | + | 147356 | 147379 | 2 | ebv-mir-BART10; | tacataaccatggagttggatgtg |
| 971 | + | 147356 | 147379 | 7 | ebv-mir-BART10; | tacataaccatggagttggctgtg |
| 972 | + | 147357 | 147374 | 31 | ebv-mir-BART10; | acataaccatggagttgg |
| 973 | + | 147357 | 147374 | 2 | ebv-mir-BART10; | acatagccatggagttgg |
| 974 | + | 147357 | 147375 | 300 | ebv-mir-BART10; | acataaccatggagttggc |
| 975 | + | 147357 | 147376 | 61 | ebv-mir-BART10; | acataaccatggagttggct |
| 976 | + | 147357 | 147377 | 25 | ebv-mir-BART10; | acataaccatggagttagctg |
| 977 | + | 147357 | 147377 | 4 | ebv-mir-BART10; | acataaccatggagttcgctg |
| 978 | + | 147357 | 147377 | 95 | ebv-mir-BART10; | acataaccatggagttggctg |
| 979 | + | 147357 | 147378 | 50 | ebv-mir-BART10; | acataaccatggagttagctgt |
| 980 | + | 147357 | 147378 | 14 | ebv-mir-BART10; | acataaccatggagttcgctgt |
| 981 | + | 147357 | 147378 | 390 | ebv-mir-BART10; | acataaccatggagttgactgt |
| 982 | + | 147357 | 147378 | 23 | ebv-mir-BART10; | acataaccatggagttgcctgt |
| 983 | + | 147357 | 147378 | 115 | ebv-mir-BART10; | acataaccatggagttggctgt |
| 984 | + | 147357 | 147378 | 11 | ebv-mir-BART10; | acataaccatggagttgtctgt |
| 985 | + | 147357 | 147379 | 4 | ebv-mir-BART10; | acataaccatggagttgactgtg |

| | | | | | | |
|---|---|---|---|---|---|---|
| 986 | + | 147357 | 147379 | 2 | ebv-mir-BART10; | acataaccatggagttggctgtg |
| 987 | + | 147357 | 147379 | 2 | ebv-mir-BART10; | acataaccatggagttggttgtg |
| 988 | + | 147358 | 147375 | 9 | ebv-mir-BART10; | cataaccatggagttggc |
| 989 | + | 147358 | 147377 | 7 | ebv-mir-BART10; | cataaccatggagttggctg |
| 990 | + | 147358 | 147378 | 10 | ebv-mir-BART10; | cataaccatggagttgactgt |
| 991 | + | 147358 | 147378 | 18 | ebv-mir-BART10; | cataaccatggagttggctgt |
| 992 | + | 147359 | 147378 | 4 | ebv-mir-BART10; | ataaccatggagttggctgt |
| 993 | + | 147359 | 147379 | 3 | ebv-mir-BART10; | ataaccatggagttggctgtg |
| 994 | + | 147361 | 147378 | 2 | ebv-mir-BART10; | aaccatggagttggctgt |
| 995 | + | 147537 | 147554 | 106 | ebv-mir-BART11; | tcagacagtttggtgcgc |
| 996 | + | 147537 | 147555 | 76 | ebv-mir-BART11; | tcagacagtttggtgcgct |
| 997 | + | 147537 | 147557 | 6 | ebv-mir-BART11; | tcagacagtttggtgcactag |
| 998 | + | 147537 | 147557 | 21 | ebv-mir-BART11; | tcagacagtttggtgcgctag |
| 999 | + | 147537 | 147558 | 37 | ebv-mir-BART11; | tcagacagtttggtgcgctagt |
| 1000 | + | 147537 | 147559 | 8 | ebv-mir-BART11; | tcagacagtttggtgcgatagtt |
| 1001 | + | 147537 | 147559 | 83 | ebv-mir-BART11; | tcagacagtttggtgcgctagtt |
| 1002 | + | 147537 | 147560 | 9 | ebv-mir-BART11; | tcagacagtttggtgcgcgagttg |
| 1003 | + | 147537 | 147560 | 3 | ebv-mir-BART11; | tcagacagtttggtgcgctagttg |
| 1004 | + | 147537 | 147561 | 3 | ebv-mir-BART11; | tcagacagtttggtgcgctagttgt |
| 1005 | + | 147537 | 147561 | 5 | ebv-mir-BART11; | tcagacagtttggtgcgctggttgt |
| 1006 | + | 147574 | 147591 | 3 | ebv-mir-BART11; | aacgcacaccaggctgac |
| 1007 | + | 147574 | 147592 | 2 | ebv-mir-BART11; | aacgcacaccaggctgact |

| | | | | | | |
|---|---|---|---|---|---|---|
| 1008 | + | 147574 | 147595 | 2 | ebv-mir-BART11; | aacgcacaccaggctgcctgcc |
| 1009 | + | 147575 | 147592 | 102 | ebv-mir-BART11; | acgcacaccaggctgact |
| 1010 | + | 147575 | 147593 | 51 | ebv-mir-BART11; | acgcacaccaggctgactg |
| 1011 | + | 147575 | 147594 | 29 | ebv-mir-BART11; | acgcacaccaggctgactgc |
| 1012 | + | 147575 | 147595 | 24 | ebv-mir-BART11; | acgcacaccaggctaactgcc |
| 1013 | + | 147575 | 147595 | 4 | ebv-mir-BART11; | acgcacaccaggctcactgcc |
| 1014 | + | 147575 | 147595 | 16 | ebv-mir-BART11; | acgcacaccaggctgaatgcc |
| 1015 | + | 147575 | 147595 | 285 | ebv-mir-BART11; | acgcacaccaggctgactgcc |
| 1016 | + | 147575 | 147595 | 25 | ebv-mir-BART11; | acgcacaccaggctgcctgcc |
| 1017 | + | 147575 | 147595 | 6 | ebv-mir-BART11; | acgcacaccaggctgtctgcc |
| 1018 | + | 147575 | 147595 | 6 | ebv-mir-BART11; | acgcacaccaggcttactgcc |
| 1019 | + | 147575 | 147595 | 2 | ebv-mir-BART11; | acgcccaccaggctgactgcc |
| 1020 | + | 147575 | 147596 | 2 | ebv-mir-BART11; | acgcacaccaggctgaatgcct |
| 1021 | + | 147575 | 147596 | 2 | ebv-mir-BART11; | acgcacaccaggctgacagcct |
| 1022 | + | 147575 | 147596 | 2 | ebv-mir-BART11; | acgcacaccaggctgaccgcct |
| 1023 | + | 147575 | 147596 | 105 | ebv-mir-BART11; | acgcacaccaggctgactgcct |
| 1024 | + | 147575 | 147596 | 3 | ebv-mir-BART11; | acgcacaccaggctgattgcct |
| 1025 | + | 147575 | 147596 | 2 | ebv-mir-BART11; | acgcataccaggctgactgcct |
| 1026 | + | 147575 | 147597 | 5 | ebv-mir-BART11; | acgcacaccaggctgactgcctt |
| 1027 | + | 147901 | 147918 | 5 | ebv-mir-BART12; | acccgcccatcaccaccg |
| 1028 | + | 147901 | 147920 | 5 | ebv-mir-BART12; | acccgcccatcaccaccgga |
| 1029 | + | 147901 | 147921 | 3 | ebv-mir-BART12; | acccgcccatcaccacaggac |

| | | | | | | |
|---|---|---|---|---|---|---|
| 1030 | + | 147901 | 147921 | 24 | ebv-mir-BART12; | acccgcccatcaccaccggac |
| 1031 | + | 147901 | 147922 | 15 | ebv-mir-BART12; | acccgcccatcaccaccggaca |
| 1032 | + | 147902 | 147920 | 2 | ebv-mir-BART12; | cccgcccatcaccaccgga |
| 1033 | + | 147936 | 147957 | 4 | ebv-mir-BART12; | tcctgtggtgtttggtgcggtt |
| 1034 | + | 147936 | 147957 | 3 | ebv-mir-BART12; | tcctgtggtgtttggtggggtt |
| 1035 | + | 147936 | 147958 | 3 | ebv-mir-BART12; | tcctgtggtgtttagtgtggttt |
| 1036 | + | 148213 | 148230 | 7 | ebv-mir-BART19; | caacattccccgcaaaca |
| 1037 | + | 148215 | 148233 | 3 | ebv-mir-BART19; | acattccccgcaaacatga |
| 1038 | + | 148215 | 148234 | 4 | ebv-mir-BART19; | acattccccgcaaacatgac |
| 1039 | + | 148215 | 148235 | 13 | ebv-mir-BART19; | acattccccgcaaacaagaca |
| 1040 | + | 148215 | 148235 | 1653 | ebv-mir-BART19; | acattccccgcaaacacgaca |
| 1041 | + | 148215 | 148235 | 25 | ebv-mir-BART19; | acattccccgcaaacaggaca |
| 1042 | + | 148215 | 148235 | 8 | ebv-mir-BART19; | acattccccgcaaacatgaca |
| 1043 | + | 148215 | 148235 | 2 | ebv-mir-BART19; | acattccccgcaaacgtgaca |
| 1044 | + | 148215 | 148236 | 246 | ebv-mir-BART19; | acattccccgcaaacaagacat |
| 1045 | + | 148215 | 148236 | 1934 | ebv-mir-BART19; | acattccccgcaaacacgacat |
| 1046 | + | 148215 | 148236 | 31 | ebv-mir-BART19; | acattccccgcaaacaggacat |
| 1047 | + | 148215 | 148236 | 5 | ebv-mir-BART19; | acattccccgcaaacatcacat |
| 1048 | + | 148215 | 148236 | 164 | ebv-mir-BART19; | acattccccgcaaacatgacat |
| 1049 | + | 148215 | 148236 | 2 | ebv-mir-BART19; | acattccccgcaaacattacat |
| 1050 | + | 148215 | 148236 | 2 | ebv-mir-BART19; | acattccccgtaaacatgacat |
| 1051 | + | 148215 | 148237 | 1074 | ebv-mir-BART19; | acattccccgcaaacacgacatg |

| | | | | | | |
|---|---|---|---|---|---|---|
| 1052 | + | 148215 | 148238 | 21 | ebv-mir-BART19; | acattccccgcaaacacgacatgg |
| 1053 | + | 148215 | 148239 | 2 | ebv-mir-BART19; | acattccccgcaaacacgacatggg |
| 1054 | + | 148216 | 148235 | 2 | ebv-mir-BART19; | cattccccgcaaacacgaca |
| 1055 | + | 148217 | 148236 | 2 | ebv-mir-BART19; | attccccgcaaacaagacat |
| 1056 | + | 148217 | 148237 | 3 | ebv-mir-BART19; | attccccgcaaacacgacatg |
| 1057 | + | 148254 | 148271 | 2 | ebv-mir-BART19; | ttttgtttgcttgggaat |
| 1058 | + | 148254 | 148273 | 2 | ebv-mir-BART19; | ttttgtttgcttgggaatgc |
| 1059 | + | 148374 | 148393 | 2 | ebv-mir-BART20; | catggaggcacagcctgtta |
| 1060 | + | 148374 | 148395 | 14 | ebv-mir-BART20; | catgaaggcacagcctattacc |
| 1061 | + | 148526 | 148543 | 21 | ebv-mir-BART13; | aaccggctcgtggctcgt |
| 1062 | + | 148526 | 148544 | 55 | ebv-mir-BART13; | aaccggctcgtggctcgta |
| 1063 | + | 148526 | 148545 | 36 | ebv-mir-BART13; | aaccggctcgtggctcgtac |
| 1064 | + | 148526 | 148546 | 2 | ebv-mir-BART13; | aaccggctcgtggctcataca |
| 1065 | + | 148526 | 148546 | 28 | ebv-mir-BART13; | aaccggctcgtggctcgtaca |
| 1066 | + | 148526 | 148546 | 2 | ebv-mir-BART13; | aaccggctcgtggcttgtaca |
| 1067 | + | 148526 | 148547 | 2 | ebv-mir-BART13; | aaccggctcgtggctcatacag |
| 1068 | + | 148526 | 148547 | 12 | ebv-mir-BART13; | aaccggctcgtggctcggacag |
| 1069 | + | 148526 | 148547 | 38 | ebv-mir-BART13; | aaccggctcgtggctcgtacag |
| 1070 | + | 148526 | 148548 | 5 | ebv-mir-BART13; | aaccggctcgtggctcgtacaga |
| 1071 | + | 148526 | 148550 | 10 | ebv-mir-BART13; | aaccggctcgtggctcgaacagacg |
| 1072 | + | 148526 | 148550 | 16 | ebv-mir-BART13; | aaccggctcgtggctcgcacagacg |
| 1073 | + | 148526 | 148550 | 13 | ebv-mir-BART13; | aaccggctcgtggctcggacagacg |

| | | | | | | |
|---|---|---|---|---|---|---|
| 1074 | + | 148526 | 148550 | 37 | ebv-mir-BART13; | aaccggctcgtggctcgtacagacg |
| 1075 | + | 148526 | 148550 | 2 | ebv-mir-BART13; | aaccggctcgtggctcgtccagacg |
| 1076 | + | 148563 | 148580 | 81 | ebv-mir-BART13; | tgtaacttgccagggacg |
| 1077 | + | 148563 | 148581 | 13 | ebv-mir-BART13; | tgtaacttgccagggacgg |
| 1078 | + | 148563 | 148582 | 14 | ebv-mir-BART13; | tgtaacttgccagggacggc |
| 1079 | + | 148563 | 148583 | 16 | ebv-mir-BART13; | tgtaacttgccagggacggct |
| 1080 | + | 148563 | 148584 | 2 | ebv-mir-BART13; | tgtaacttgccagggaccgctg |
| 1081 | + | 148563 | 148584 | 20 | ebv-mir-BART13; | tgtaacttgccagggacggctg |
| 1082 | + | 148563 | 148584 | 2 | ebv-mir-BART13; | tgtaacttgccagggactgctg |
| 1083 | + | 148563 | 148585 | 3 | ebv-mir-BART13; | tgtaacttgccagggacgactga |
| 1084 | + | 148563 | 148585 | 45 | ebv-mir-BART13; | tgtaacttgccagggacggctga |
| 1085 | + | 148563 | 148586 | 2 | ebv-mir-BART13; | tgtaacttgccagggacagctgac |
| 1086 | + | 148563 | 148586 | 6 | ebv-mir-BART13; | tgtaacttgccagggacgactgac |
| 1087 | + | 148563 | 148586 | 3 | ebv-mir-BART13; | tgtaacttgccagggacggctgac |
| 1088 | + | 148563 | 148587 | 19 | ebv-mir-BART13; | tgtaacttgccagggacagctgacg |
| 1089 | + | 148563 | 148587 | 59 | ebv-mir-BART13; | tgtaacttgccagggacgactgacg |
| 1090 | + | 148563 | 148587 | 5 | ebv-mir-BART13; | tgtaacttgccagggacgcctgacg |
| 1091 | + | 148563 | 148587 | 4 | ebv-mir-BART13; | tgtaacttgccagggacggatgacg |
| 1092 | + | 148563 | 148587 | 23 | ebv-mir-BART13; | tgtaacttgccagggacggcagacg |
| 1093 | + | 148563 | 148587 | 21 | ebv-mir-BART13; | tgtaacttgccagggacggctgacg |
| 1094 | + | 148744 | 148761 | 26 | ebv-mir-BART14; | taccctacgctgccgatt |
| 1095 | + | 148744 | 148762 | 4 | ebv-mir-BART14; | taccctacgctgccgattt |

| | | | | | | |
|---|---|---|---|---|---|---|
| 1096 | + | 148744 | 148764 | 2 | ebv-mir-BART14; | taccctacgctgccgaattac |
| 1097 | + | 148744 | 148764 | 4 | ebv-mir-BART14; | taccctacgctgccgagttac |
| 1098 | + | 148744 | 148764 | 66 | ebv-mir-BART14; | taccctacgctgccgatttac |
| 1099 | + | 148744 | 148764 | 13 | ebv-mir-BART14; | taccctacgctgccggtttac |
| 1100 | + | 148744 | 148765 | 2 | ebv-mir-BART14; | taccctacgctgccgaattaca |
| 1101 | + | 148744 | 148765 | 3 | ebv-mir-BART14; | taccctacgctgccgacttaca |
| 1102 | + | 148744 | 148765 | 6 | ebv-mir-BART14; | taccctacgctgccgagttaca |
| 1103 | + | 148744 | 148765 | 4 | ebv-mir-BART14; | taccctacgctgccgatataca |
| 1104 | + | 148744 | 148765 | 51 | ebv-mir-BART14; | taccctacgctgccgatctaca |
| 1105 | + | 148744 | 148765 | 2 | ebv-mir-BART14; | taccctacgctgccgatgtaca |
| 1106 | + | 148744 | 148765 | 25 | ebv-mir-BART14; | taccctacgctgccgatttaca |
| 1107 | + | 148744 | 148766 | 2 | ebv-mir-BART14; | taccctacgctgccgatttacat |
| 1108 | + | 148745 | 148762 | 2 | ebv-mir-BART14; | accctacgctgccgattt |
| 1109 | + | 148745 | 148763 | 4 | ebv-mir-BART14; | accctacgctgccgattta |
| 1110 | + | 148745 | 148764 | 2 | ebv-mir-BART14; | accctacgctgccgagttac |
| 1111 | + | 148745 | 148764 | 18 | ebv-mir-BART14; | accctacgctgccgatttac |
| 1112 | + | 148745 | 148765 | 8 | ebv-mir-BART14; | accctacgctgccgatttaca |
| 1113 | + | 148746 | 148765 | 4 | ebv-mir-BART14; | ccctacgctgccgatttaca |
| 1114 | + | 148778 | 148796 | 13 | ebv-mir-BART14; | taaatgctgcagtagtagg |
| 1115 | + | 148778 | 148797 | 4 | ebv-mir-BART14; | taaatgctgcagtagtaggg |
| 1116 | + | 148778 | 148798 | 2 | ebv-mir-BART14; | taaatgctgcagtaggaggga |
| 1117 | + | 148778 | 148798 | 20 | ebv-mir-BART14; | taaatgctgcagtagtaggga |

| | | | | | | |
|---|---|---|---|---|---|---|
| 1118 | + | 148778 | 148799 | 2 | ebv-mir-BART14; | taaatgctgcagtagtaaggat |
| 1119 | + | 148778 | 148799 | 10 | ebv-mir-BART14; | taaatgctgcagtagtagggat |
| 1120 | + | 148778 | 148800 | 2 | ebv-mir-BART14; | taaatgctgcagtagtagagatc |
| 1121 | + | 148778 | 148800 | 23 | ebv-mir-BART14; | taaatgctgcagtagtagggatc |
| 1122 | + | 148778 | 148801 | 3 | ebv-mir-BART14; | taaatgctgcagtagtagggatct |
| 1123 | + | 148779 | 148800 | 10 | ebv-mir-BART14; | aaatgctgcagtagttgggatc |
| 1124 | + | 148801 | 148825 | 2 | - | tggacgcgcgacctgctactcttcg |
| 1125 | + | 150266 | 150285 | 6 | - | aacgacgagcgccgggacgc |
| 1126 | + | 152747 | 152765 | 3 | ebv-mir-BART2; | tattttctgcattcaccct |
| 1127 | + | 152747 | 152766 | 2 | ebv-mir-BART2; | tattttctgcattcaccctt |
| 1128 | + | 152747 | 152767 | 18 | ebv-mir-BART2; | tattttctgcattcgcacttg |
| 1129 | + | 152747 | 152767 | 5 | ebv-mir-BART2; | tattttctgcattcgcgcttg |
| 1130 | + | 152747 | 152767 | 8 | ebv-mir-BART2; | tattttctgcattcgctcttg |
| 1131 | + | 152747 | 152768 | 2 | ebv-mir-BART2; | tattttctgaattcgcccttgc |
| 1132 | + | 152747 | 152768 | 31 | ebv-mir-BART2; | tattttctgcattcgcacttgc |
| 1133 | + | 152747 | 152768 | 5 | ebv-mir-BART2; | tattttctgcattcgccattgc |
| 1134 | + | 152747 | 152768 | 8 | ebv-mir-BART2; | tattttctgcattcgccgttgc |
| 1135 | + | 152747 | 152768 | 9 | ebv-mir-BART2; | tattttctgcattcgcgcttgc |
| 1136 | + | 152747 | 152768 | 25 | ebv-mir-BART2; | tattttctgcattcgctcttgc |
| 1137 | + | 152747 | 152769 | 39 | ebv-mir-BART2; | tattttctgcattcgaccttgcg |
| 1138 | + | 152747 | 152769 | 3 | ebv-mir-BART2; | tattttctgcattcgcacttgcg |
| 1139 | + | 152747 | 152769 | 2 | ebv-mir-BART2; | tattttctgcattcgcgcttgcg |

| | | | | | | |
|---|---|---|---|---|---|---|
| 1140 | + | 152747 | 152769 | 13 | ebv-mir-BART2; | tattttctgcattcgtccttgcg |
| 1141 | + | 152783 | 152800 | 7 | ebv-mir-BART2; | aaggagcgatttggagaa |
| 1142 | + | 152807 | 152831 | 2 | - | ctgtgagtttcacagatccacgggc |
| 1143 | + | 152867 | 152884 | 2 | - | ttcccgatgatgatgaca |
| 1144 | + | 152867 | 152889 | 5 | - | ttcccgatgatgatgacaaccgc |
| 1145 | + | 152868 | 152885 | 13 | - | tcccgatgatgatgacaa |
| 1146 | + | 152868 | 152886 | 30 | - | tcccgatgatgatgacaac |
| 1147 | + | 152868 | 152886 | 4 | - | tcccggtgatgatgacaac |
| 1148 | + | 152868 | 152887 | 24 | - | tcccgatgatgatgacaacc |
| 1149 | + | 152868 | 152888 | 29 | - | tcccgatgatgatgacaaccg |
| 1150 | + | 152868 | 152889 | 8 | - | tcccgatgatgataacaaccgc |
| 1151 | + | 152868 | 152889 | 340 | - | tcccgatgatgatgacaaccgc |
| 1152 | + | 152868 | 152889 | 11 | - | tcccgatgatgatgacacccgc |
| 1153 | + | 152868 | 152889 | 6 | - | tcccgatgatgatgacatccgc |
| 1154 | + | 152868 | 152889 | 2 | - | tcccgatgatgatgacgaccgc |
| 1155 | + | 152868 | 152889 | 6 | - | tcccgatgatgatggcaaccgc |
| 1156 | + | 152868 | 152889 | 2 | - | tcccgatgatgattacaaccgc |
| 1157 | + | 152868 | 152889 | 2 | - | tcccgatgatggtgacaaccgc |
| 1158 | + | 152868 | 152890 | 67 | - | tcccgatgatgatgacaaccgcg |
| 1159 | + | 152868 | 152890 | 2 | - | tcccgatgatgatgataaccgcg |
| 1160 | + | 152868 | 152891 | 9 | - | tcccgatgatgatgacaaccgcgg |
| 1161 | + | 152868 | 152892 | 509 | - | tcccgatgatgatgacaaccgcggc |

| | | | | | | |
|---|---|---|---|---|---|---|
| 1162 | + | 152869 | 152886 | 2 | - | cccgatgatgacgacaac |
| 1163 | + | 152869 | 152886 | 115 | - | cccgatgatgatgacaac |
| 1164 | + | 152869 | 152887 | 12 | - | cccgatgatgatgaaaacc |
| 1165 | + | 152869 | 152887 | 69 | - | cccgatgatgatgacaacc |
| 1166 | + | 152869 | 152888 | 82 | - | cccgatgatgatgacaaccg |
| 1167 | + | 152869 | 152889 | 40 | - | cccgatgatgataacaaccgc |
| 1168 | + | 152869 | 152889 | 3 | - | cccgatgatgatcacaaccgc |
| 1169 | + | 152869 | 152889 | 4 | - | cccgatgatgatgaaaaccgc |
| 1170 | + | 152869 | 152889 | 616 | - | cccgatgatgatgacaaccgc |
| 1171 | + | 152869 | 152889 | 8 | - | cccgatgatgatgacacccgc |
| 1172 | + | 152869 | 152889 | 5 | - | cccgatgatgatgacagccgc |
| 1173 | + | 152869 | 152889 | 3 | - | cccgatgatgatgataaccgc |
| 1174 | + | 152869 | 152889 | 5 | - | cccgatgatgatggcaaccgc |
| 1175 | + | 152869 | 152889 | 3 | - | cccgatgatgatgtcaaccgc |
| 1176 | + | 152869 | 152890 | 209 | - | cccgatgatgatgacaaccgcg |
| 1177 | + | 152869 | 152890 | 2 | - | cccgatgatgatgacacccgcg |
| 1178 | + | 152869 | 152890 | 7 | - | cccgatgatgatgacgaccgcg |
| 1179 | + | 152869 | 152890 | 7 | - | cccgatgatgatgataaccgcg |
| 1180 | + | 152869 | 152891 | 112 | - | cccgatgatgatgacaaccgcgg |
| 1181 | + | 152869 | 152892 | 3 | - | cccgatgatgatgacaaccacggc |
| 1182 | + | 152869 | 152892 | 110 | - | cccgatgatgatgacaaccgcggc |
| 1183 | + | 152869 | 152893 | 2 | - | cccgatgatgataacaaccgcggct |

| | | | | | | |
|---|---|---|---|---|---|---|
| 1184 | + | 152869 | 152893 | 1723 | - | cccgatgatgatgacaaccgcggct |
| 1185 | + | 152869 | 152893 | 4 | - | cccggtgatgatgacaaccgcggct |
| 1186 | + | 152869 | 152893 | 2 | - | cccgttgatgatgacaaccgcggct |
| 1187 | + | 152872 | 152889 | 30 | - | gatgatgatgacaaccgc |
| 1188 | + | 152872 | 152889 | 3 | - | gatgatgatgacgaccgc |
| 1189 | + | 152872 | 152890 | 3 | - | gatgatgatgacaaccgcg |
| 1190 | + | 152872 | 152892 | 2 | - | gatgatgatgacaaccgcggc |
| 1191 | + | 152872 | 152896 | 26 | - | gatgatgatgacaaccgcggctgtc |
| 1192 | + | 152900 | 152918 | 2 | - | agcggctgacgaaatcggt |
| 1193 | + | 152900 | 152919 | 2 | - | agcggctgacgaaatcggtt |
| 1194 | + | 152900 | 152924 | 7 | - | agcggctgacgaaatcggttgagat |
| 1195 | + | 152901 | 152919 | 4 | - | gcggctgacgaaatcggtt |
| 1196 | + | 152901 | 152924 | 2 | - | gcggctgacgaaatcggttgagat |
| 1197 | + | 152901 | 152925 | 11 | - | gcggctgacgaaatcggttgagatt |
| 1198 | + | 152902 | 152926 | 10 | - | cggctgacgaaatcggttgagattc |
| 1199 | + | 152906 | 152929 | 4 | - | tgacgaaatcggttgagattctga |
| 1200 | + | 155613 | 155631 | 2 | - | acccgtgcttgtcttgcag |
| 1201 | + | 156200 | 156224 | 2 | - | ggaatgagacgcaggtacgccttgt |
| 1202 | + | 157314 | 157331 | 3 | - | cttggctgccaccgcctt |
| 1203 | + | 159705 | 159722 | 5 | YP_401716.1; | acgcctcctccccgcctc |
| 1204 | + | 159707 | 159726 | 2 | YP_401716.1; | gcctcctcctcgccttggcc |
| 1205 | + | 160630 | 160647 | 57 | - | cgggaaccgggtaccctt |

| | | | | | | |
|---|---|---|---|---|---|---|
| 1206 | + | 160630 | 160647 | 2 | - | cgggacacgggtaccctt |
| 1207 | + | 160630 | 160648 | 21 | - | cgggaaccgggtacccttt |
| 1208 | + | 161781 | 161798 | 2 | - | ggtctagccgcacgcctt |
| 1209 | + | 163533 | 163551 | 4 | - | tgaggctgggaacgccttg |
| 1210 | + | 163998 | 164015 | 2 | - | cccctcaaagatgcgggg |
| 1211 | + | 163998 | 164017 | 2 | - | cccctcaaagatgcggggaa |
| 1212 | + | 167791 | 167813 | 3 | - | gtggatcaccgccgccttggccg |
| 1213 | + | 167792 | 167813 | 2 | - | tggatcaccgccgccttggccg |
| 1214 | + | 167793 | 167813 | 11 | - | ggatcaccgccgccttggccg |
| 1215 | + | 169380 | 169398 | 81 | - | aggcgcggggcgcggaacg |
| 1216 | + | 170692 | 170709 | 2 | - | ccccttggggcatggggg |
| 1217 | + | 170692 | 170710 | 2 | - | ccccttggggcatgggggg |
| 1218 | - | 2699 | 2719 | 2 | - | gcgccgggccgccgccttggc |
| 1219 | - | 38189 | 38213 | 2 | - | acactctaaaagtaacctgtctact |
| 1220 | - | 40317 | 40334 | 5 | - | cggtcctcccccgtgaac |
| 1221 | - | 46096 | 46115 | 3 | YP_401648.1; | gatggaggactttgggccct |
| 1222 | - | 53020 | 53038 | 3 | YP_401652.1; | accccacccggacgccccc |
| 1223 | - | 53046 | 53063 | 3 | YP_401652.1; | ggagaacccagacgcctt |
| 1224 | - | 53897 | 53917 | 8 | YP_401652.1; | cccctcgaggccgccttggcc |
| 1225 | - | 56924 | 56942 | 11 | YP_401652.1; | gccagaggcagacgccttg |
| 1226 | - | 57523 | 57540 | 2 | YP_401652.1; | ccccggctccttcaccac |
| 1227 | - | 57574 | 57591 | 2 | YP_401652.1; | ccccggctccttcaccac |

| | | | | | | |
|---|---|---|---|---|---|---|
| 1228 | - | 62380 | 62397 | 2 | YP_401653.1; | ggccgggtgggacgcctt |
| 1229 | - | 65955 | 65972 | 5 | - | cttggtggacgccttggc |
| 1230 | - | 67221 | 67241 | 2 | - | ccttggtcggtccgccttggc |
| 1231 | - | 74337 | 74354 | 2 | YP_401662.1; | ggaggaggccttcgcctt |
| 1232 | - | 76569 | 76587 | 2 | - | cccctttaaaaatcccca |
| 1233 | - | 77004 | 77021 | 7 | - | cagggctgggttcgcctt |
| 1234 | - | 80677 | 80694 | 2 | - | taaaggggccggcggaaa |
| 1235 | - | 86700 | 86717 | 2 | - | tgctgtacgatattccat |
| 1236 | - | 95591 | 95615 | 3 | - | cagtgaatacagggagtgcagactc |
| 1237 | - | 98878 | 98895 | 2 | - | cgcctgtccctgcaagac |
| 1238 | - | 110163 | 110180 | 2 | YP_401688.1; | cagtggtgccggcgcctt |
| 1239 | - | 118794 | 118811 | 3 | - | aagaggccgggacgcctt |
| 1240 | - | 119984 | 120001 | 4 | YP_401695.1; | gggagagggtgtaagtct |
| 1241 | - | 121383 | 121400 | 2 | YP_401697.1; | gccaggcggccgtcccgg |
| 1242 | - | 121556 | 121575 | 3 | YP_401697.1; | ccaaggcgacgggccgcctg |
| 1243 | - | 121557 | 121574 | 196 | YP_401697.1; | caaggcgacgggccgcct |
| 1244 | - | 121557 | 121575 | 300 | YP_401697.1; | ccaaggcgacgggccgcct |
| 1245 | - | 121557 | 121575 | 2 | YP_401697.1; | ccaagggaacgggccgcct |
| 1246 | - | 134299 | 134317 | 3 | - | ccgggtggccgccttggcc |
| 1247 | - | 134968 | 134987 | 7 | YP_401726.1; | gcctctggcacgccttggcc |
| 1248 | - | 136575 | 136592 | 2 | - | aaaagggggggcaacgggg |
| 1249 | - | 136575 | 136592 | 14 | - | aaaagggggggccgcgggg |

| | | | | | | |
|---|---|---|---|---|---|---|
| 1250 | - | 138026 | 138044 | 2 | YP_401706.1; | cccttggccgttgctactt |
| 1251 | - | 138026 | 138045 | 37 | YP_401706.1; | gcccttggccgtacctactt |
| 1252 | - | 138026 | 138045 | 2 | YP_401706.1; | gcccttggccgttgctactt |
| 1253 | - | 138028 | 138045 | 4 | YP_401706.1; | gcccttggccgtacctac |
| 1254 | - | 139196 | 139213 | 10 | - | agagcgcccccccccc |
| 1255 | - | 140392 | 140409 | 7 | - | agacccccaacgccttgg |
| 1256 | - | 140849 | 140866 | 2 | YP_401707.1; | ccggaaccccggccccgg |
| 1257 | - | 140849 | 140866 | 11 | YP_401707.1; | ccggaaccccgtacccgg |
| 1258 | - | 140951 | 140968 | 2 | YP_401707.1; | ccggaaccccggccccgg |
| 1259 | - | 140951 | 140968 | 11 | YP_401707.1; | ccggaaccccgtacccgg |
| 1260 | - | 141053 | 141070 | 2 | YP_401707.1; | ccggaaccccggccccgg |
| 1261 | - | 141053 | 141070 | 11 | YP_401707.1; | ccggaaccccgtacccgg |
| 1262 | - | 141155 | 141172 | 2 | YP_401707.1; | ccggaaccccggccccgg |
| 1263 | - | 141155 | 141172 | 11 | YP_401707.1; | ccggaaccccgtacccgg |
| 1264 | - | 141257 | 141274 | 2 | YP_401707.1; | ccggaaccccggccccgg |
| 1265 | - | 141257 | 141274 | 11 | YP_401707.1; | ccggaaccccgtacccgg |
| 1266 | - | 141359 | 141376 | 2 | YP_401707.1; | ccggaaccccggccccgg |
| 1267 | - | 141359 | 141376 | 11 | YP_401707.1; | ccggaaccccgtacccgg |
| 1268 | - | 141461 | 141478 | 2 | YP_401707.1; | ccggaaccccggccccgg |
| 1269 | - | 141461 | 141478 | 11 | YP_401707.1; | ccggaaccccgtacccgg |
| 1270 | - | 141563 | 141580 | 2 | YP_401707.1; | ccggaaccccggccccgg |
| 1271 | - | 141563 | 141580 | 11 | YP_401707.1; | ccggaaccccgtacccgg |

| | | | | | | |
|---|---|---|---|---|---|---|
| 1272 | - | 141665 | 141682 | 2 | YP_401707.1; | ccggaaccccggccccgg |
| 1273 | - | 141665 | 141682 | 11 | YP_401707.1; | ccggaaccccgtacccgg |
| 1274 | - | 141767 | 141784 | 2 | YP_401707.1; | ccggaaccccggccccgg |
| 1275 | - | 141767 | 141784 | 11 | YP_401707.1; | ccggaaccccgtacccgg |
| 1276 | - | 141869 | 141886 | 2 | YP_401707.1; | ccggaaccccggccccgg |
| 1277 | - | 141869 | 141886 | 11 | YP_401707.1; | ccggaaccccgtacccgg |
| 1278 | - | 141971 | 141988 | 2 | YP_401707.1; | ccggaaccccggccccgg |
| 1279 | - | 141971 | 141988 | 11 | YP_401707.1; | ccggaaccccgtacccgg |
| 1280 | - | 142073 | 142090 | 2 | YP_401707.1; | ccggaaccccggccccgg |
| 1281 | - | 142073 | 142090 | 11 | YP_401707.1; | ccggaaccccgtacccgg |
| 1282 | - | 142175 | 142192 | 2 | YP_401707.1; | ccggaaccccggccccgg |
| 1283 | - | 142175 | 142192 | 11 | YP_401707.1; | ccggaaccccgtacccgg |
| 1284 | - | 142277 | 142294 | 2 | YP_401707.1; | ccggaaccccggccccgg |
| 1285 | - | 142277 | 142294 | 11 | YP_401707.1; | ccggaaccccgtacccgg |
| 1286 | - | 142379 | 142396 | 2 | YP_401707.1; | ccggaaccccggccccgg |
| 1287 | - | 142379 | 142396 | 11 | YP_401707.1; | ccggaaccccgtacccgg |
| 1288 | - | 142481 | 142498 | 2 | YP_401707.1; | ccggaaccccggccccgg |
| 1289 | - | 142481 | 142498 | 11 | YP_401707.1; | ccggaaccccgtacccgg |
| 1290 | - | 142583 | 142600 | 2 | YP_401707.1; | ccggaaccccggccccgg |
| 1291 | - | 142583 | 142600 | 11 | YP_401707.1; | ccggaaccccgtacccgg |
| 1292 | - | 142685 | 142702 | 2 | YP_401707.1; | ccggaaccccggccccgg |
| 1293 | - | 142685 | 142702 | 11 | YP_401707.1; | ccggaaccccgtacccgg |

| | | | | | | |
|---|---|---|---|---|---|---|
| 1294 | - | 142787 | 142804 | 2 | YP_401707.1; | ccggaaccccggccccgg |
| 1295 | - | 142787 | 142804 | 11 | YP_401707.1; | ccggaaccccgtacccgg |
| 1296 | - | 142889 | 142906 | 2 | YP_401707.1; | ccggaaccccggccccgg |
| 1297 | - | 142889 | 142906 | 11 | YP_401707.1; | ccggaaccccgtacccgg |
| 1298 | - | 142991 | 143008 | 2 | YP_401707.1; | ccggaaccccggccccgg |
| 1299 | - | 142991 | 143008 | 11 | YP_401707.1; | ccggaaccccgtacccgg |
| 1300 | - | 143093 | 143110 | 2 | YP_401707.1; | ccggaaccccggccccgg |
| 1301 | - | 143093 | 143110 | 11 | YP_401707.1; | ccggaaccccgtacccgg |
| 1302 | - | 143195 | 143212 | 2 | YP_401707.1; | ccggaaccccggccccgg |
| 1303 | - | 143195 | 143212 | 11 | YP_401707.1; | ccggaaccccgtacccgg |
| 1304 | - | 154071 | 154088 | 6 | YP_401712.1; | cccccaccccggacgcct |
| 1305 | - | 154071 | 154088 | 20 | YP_401712.1; | ccccctccccggacgcct |
| 1306 | - | 157846 | 157863 | 3 | YP_401713.1; | agagcgcccggacgcctt |
| 1307 | - | 158946 | 158963 | 6 | YP_401715.1; | cgtacgactcttaccgcc |
| 1308 | - | 169998 | 170015 | 3 | - | gctccccgcccgccttgg |
| 1309 | - | 169999 | 170018 | 8 | - | cccgctccccgcccgccttg |
| 1310 | - | 170536 | 170553 | 3 | - | gctccccgcccgccttgg |
| 1311 | - | 170537 | 170556 | 8 | - | cccgctccccgcccgccttg |
| 1312 | - | 171059 | 171076 | 3 | - | gctccccgcccgccttgg |
| 1313 | - | 171060 | 171079 | 8 | - | cccgctccccgcccgccttg |
| 1314 | - | 171597 | 171614 | 3 | - | gctccccgcccgccttgg |
| 1315 | - | 171598 | 171617 | 8 | - | cccgctccccgcccgccttg |
