# Supplementary figures and images for: UMARS: Un-MAppable Reads Solution
Source: BMC Bioinformatics. 2011 Feb 15;12(Suppl 1):S9. doi: 10.1186/1471-2105-12-S1-S9 (PMC3044317; doi:10.1186/1471-2105-12-S1-S9)

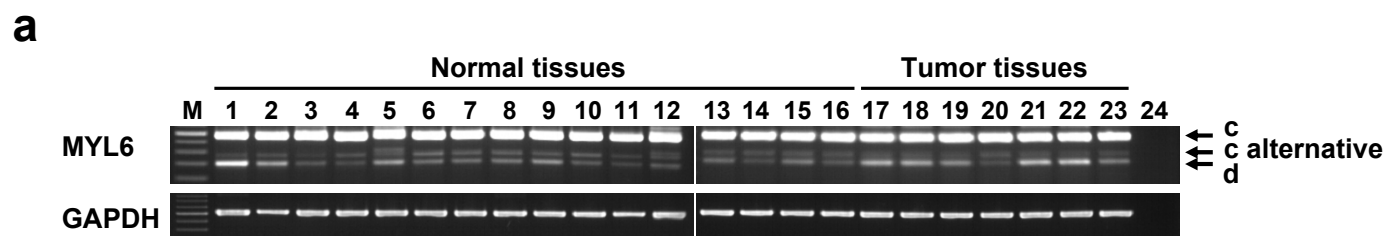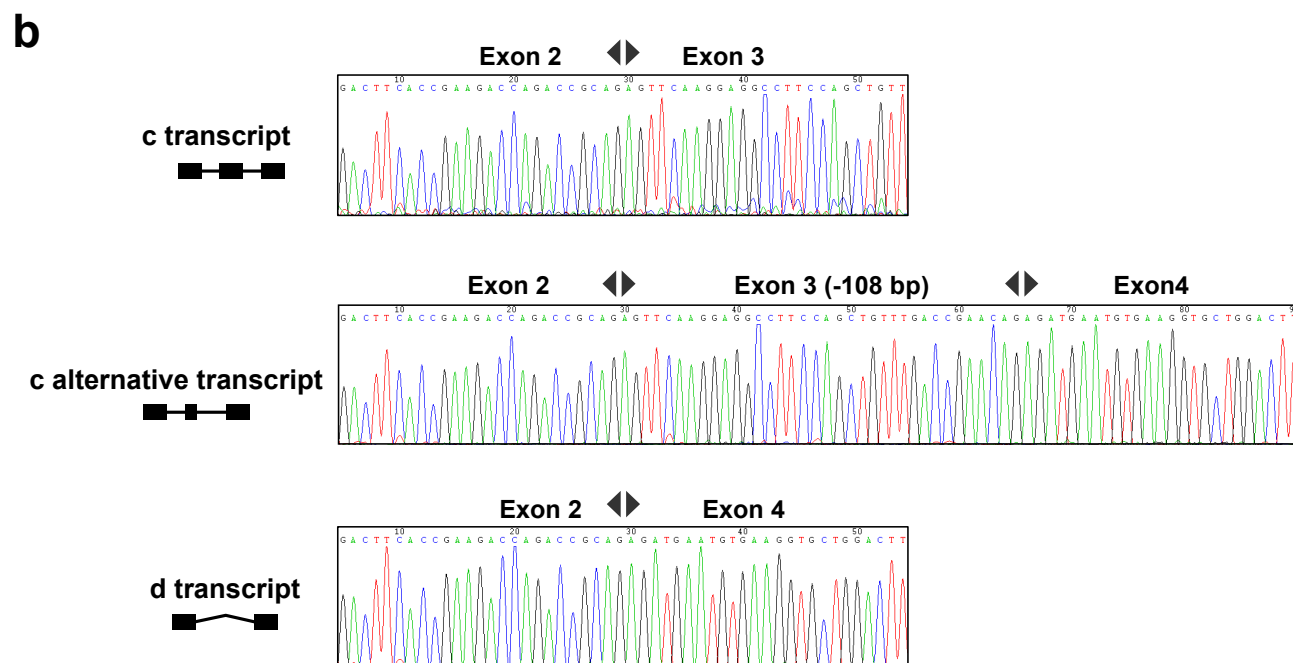

Supplement: Additional file 6 — PCR experimental validation of the detected EEJ, NM_021019 (7:2-4). The description of the marker lane and the abbreviations are the same with the ones of Fig. 4c. The forward and reverse primers were located at exon 2 and exon 4, respectively. (a) The PCR result showed that the expected EEJ (d transcript) can be experimentally detected. Besides, the major c and the minor d transcripts, the c alternative transcript (with 108 bp fewer than c transcript) was also detected. Both of the detected c alternative and d transcript have EST evidences. (b) The sequencing result provided the authenticity of the detected EEJs. [file 1471-2105-12-S1-S9-S6.pdf]

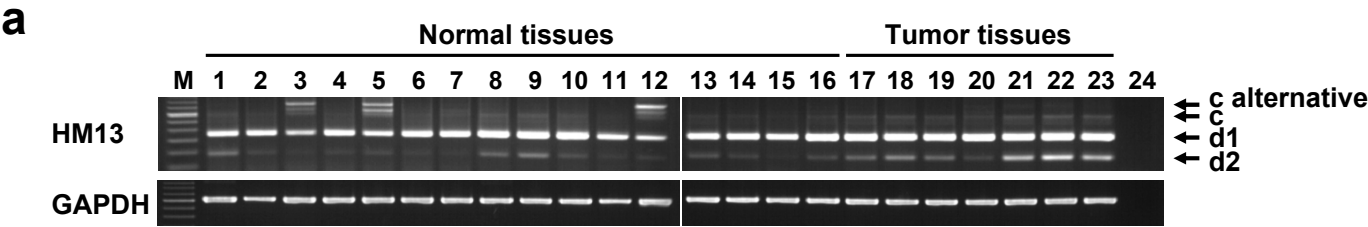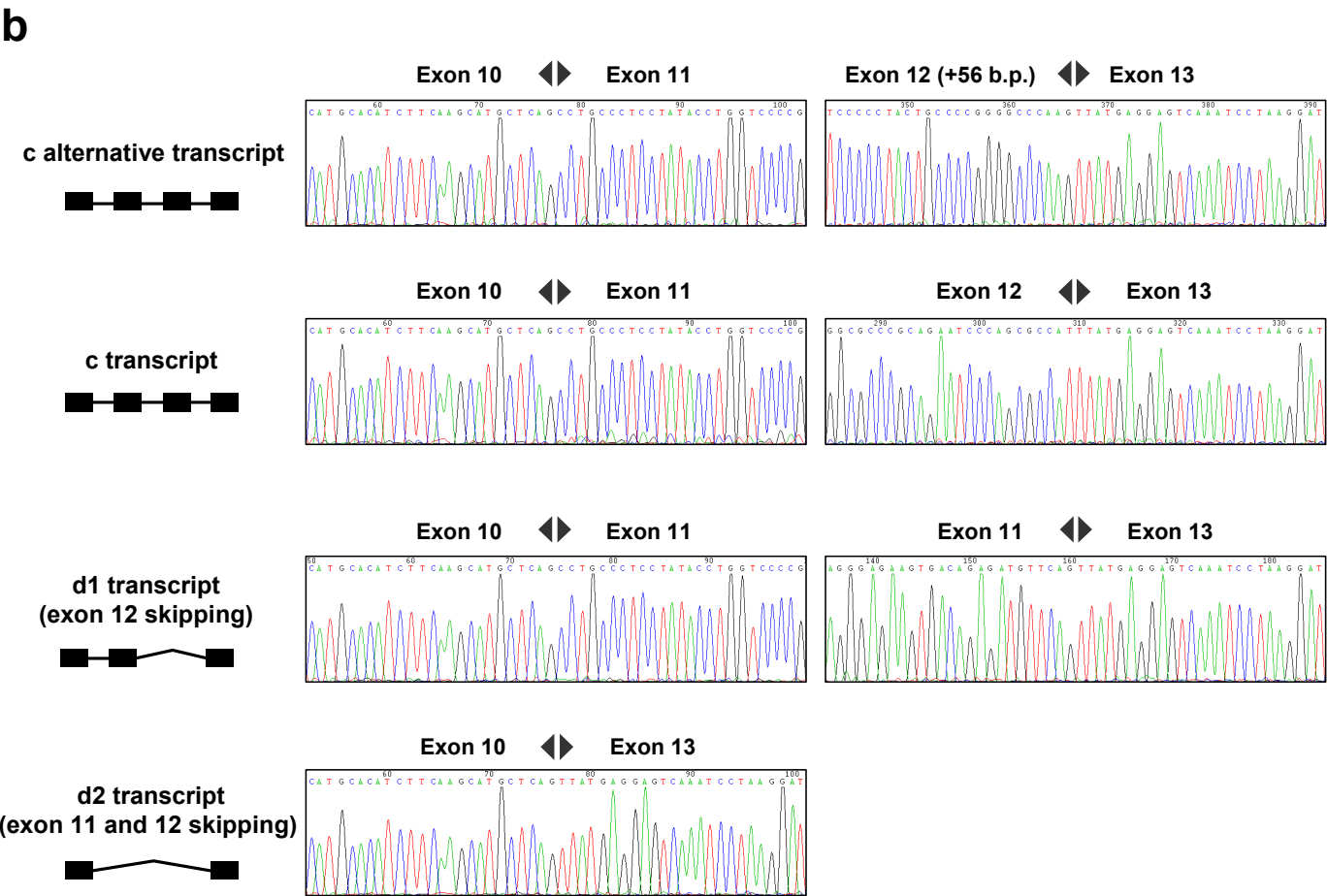

Supplement: Additional file 7 — PCR experimental validation of the detected EEJ, NM_178580 (13:10-13). The description of the marker lane and the abbreviations are the same with the ones of Fig. 4c. The forward and reverse primers were located at exon 10 and exon 13, respectively. (a) The PCR result showed that the expected EEJ (d2 transcript) can be experimentally detected. A c alternative transcript (with 56 bp more than c transcript) was also detected. In addition the d2 transcript (with exon 11 and 12 skiping), we also detected a d1 transcript (with exon 12 skipping, not originally detected by UMARS). (b) The sequencing result provided the authenticity of the detected EEJs. [file 1471-2105-12-S1-S9-S7.pdf]
